# Green/red fluorescent protein disrupting drugs for real‐time permeability tracking in three‐dimensional tumor spheroids

**DOI:** 10.1002/btm2.10731

**Published:** 2024-12-09

**Authors:** Maytal Avrashami, Danna Niezni, Dana Meron Azagury, Hagit Sason, Yosi Shamay

**Affiliations:** ^1^ Faculty of Biomedical Engineering Technion – Israel Institute of Technology Haifa Israel

**Keywords:** covalent inhibitors, drug penetration, fluorescent proteins, tumor spheroids

## Abstract

Three‐dimensional (3D) spheroid models offer a more physiologically relevant and complex environment compared to traditional two‐dimensional cultures, making them a promising tool for studying tumor biology and drug response. However, these models often face challenges in real‐time monitoring of drug diffusion, penetration, and target engagement, limiting their predictive power for in vivo and clinical outcomes. This study introduces a novel approach for real‐time tracking of drug permeability using small molecule drugs with GFP/RFP‐disrupting properties that correlate with their efficacy. We developed a reproducible 3D spheroid model with various cancer cell lines expressing GFP/RFP for efficient drug screening. Through screening over 20 FDA‐approved enzyme inhibitors, we identified three covalent kinase inhibitors—osimertinib, afatinib, and neratinib—that irreversibly disrupt GFP and RFP fluorescence. Our results reveal distinct drug diffusion and penetration profiles within GFP/RFP‐expressing spheroids, varying with drug concentration and formulation, and correlating with clinical volume of distribution (Vd) values. Additionally, we demonstrate that our approach is useful for evaluating different drug formulations as well as screening penetration enhancers for solid tumors. These findings offer a valuable 3D model for studying kinetics of drug permeability and efficacy in tumor‐like environments, with potential implications for drug delivery research and formulation development.

**Translational Impact Statement**

Current three‐dimensional (3D) tumor models struggle with real‐time monitoring of drug behavior. Using a reproducible 3D spheroid system we established, we screened FDA‐approved enzyme inhibitors to discover drugs that can disrupt fluorescence. We discovered three GFP/RFP‐disrupting molecules, enabling real‐time tracking of drug diffusion and penetration. This innovative approach provides insights into distinct drug diffusion profiles, correlating with clinical trial data. The disruptive drugs, coupled with the 3D spheroid model, can enhance drug delivery research, improving our understanding of drug diffusion in solid tumors for more effective cancer therapies.

## 1 INTRODUCTION

Understanding drug permeability in solid tumors and the ability to manipulate it are critical concepts in cancer pharmacology and therapeutics.^1^ The efficacy of treatment relies heavily on the drug's ability to penetrate and distribute effectively within these tumors.^2^ Suboptimal drug delivery can interfere with the treatment outcomes and foster drug resistance, highlighting the urgent need to develop methods that improve drug permeability and allow for precise monitoring of drug distribution.^1^, ^3^, ^4^

Three‐dimensional (3D) models have revolutionized cancer research by offering a more realistic simulation of the in vivo tumor environment than traditional two‐dimensional (2D) models.^5^, ^6^ Yet, while they advanced the understanding of tumor biology, these models currently fall short in providing real‐time, continuous data on drug diffusion and penetration in tumors.^7^ The existing tools for characterization include sectioning or the utilization of fluorescence‐based imaging using fluorescent probes or fluorescent drugs. Nevertheless, these imaging techniques encounter challenges such as laborious sample preparation, high photobleaching, and slow data acquisition speed.^6^

The use of fluorescent drugs such as doxorubicin and their associated nanoparticles presents a unique set of challenges. Real‐time, live imaging is limited by the need for extensive washing procedures to eliminate excess unabsorbed fluorescent material.^8^ These washing procedures are particularly damaging to 3D spheroids, which require low attachment conditions to maintain their structure, thereby limiting their utility in large‐scale experiments.^3^, ^9^

A conventional method for visualizing drug target engagement involves using activatable fluorescent peptide probes designed to emit light upon enzymatic degradation.^10^, ^11^ However, while this technique is suitable for enzyme targets, these probes do not provide an accurate reflection of small molecule drug dynamics due to their size and distinct physicochemical properties.^12^ Furthermore, these probes primarily function in extracellular or lysosomal proteases, limiting their range of application.^4^

Searching for more versatile and effective methods for real‐time, high‐throughput monitoring of drug delivery, penetration, and engagement, we focused our attention on green/red fluorescent proteins (GFP/RFP) and their inhibition with small molecule drugs. Despite the extensive use of fluorescent proteins in biological and biomedical research,^13^, ^14^ there are no known small molecule drug‐like inhibitors or disruptors of GFP/RFP fluorescence. We hypothesize that such a disruptor could serve as a valuable tool for visualizing and quantifying drug penetration and diffusion in real‐time, circumventing some of the existing limitations of current models and techniques.^15^ This significant gap in the current knowledge is surprising as GFP has been studied for more than 30 years and is one of the most popular research tools in biomedicine.

In this study, we aim to bridge the identified gap by conducting a screening, using automated microscopy, of more than 20 small molecule enzyme inhibitors for their potential to disrupt GFP/RFP. Our investigation led to the discovery that osimertinib, neratinib, and afatinib, covalent inhibitors of epidermal growth factor receptor (EGFR) with an acrylamide warhead,^16^ can irreversibly inhibit different types of GFP and RFP fluorescence at micromolar concentrations, in direct correlation with their efficacy. Intriguingly, we observed varying kinetic profiles for these drugs, suggesting the existence of distinct mechanisms of action for the permeability and activity of each compound. We successfully correlated drug permeability profiles in high serum with reported clinical volume of distribution (Vd) values with experiments, and demonstrated that these GFP/RFP disrupting drugs could serve as a tool to visualize the kinetic differences in nanoprecipitation based formulation, a cutting‐edge area in drug delivery research. These compounds can also assist in identifying and studying penetration enhancers, an important aspect of improving drug distribution within solid tumors.

Our findings represent a significant advancement in the quest for effective real‐time monitoring of drug delivery within solid tumors. This could improve the development of novel formulations, drug combinations, and insight into cancer therapies for solid tumors.

## 2 RESULTS

### 2.1 Discovering green fluorescent protein disrupting drugs

Efficient drug penetration into solid tumors is essential for successful treatments. Hence, we sought to find GFP/RFP inhibitors via high‐throughput 3D spheroid model to evaluate the permeability of small molecule drugs into solid tumors. We started by testing the effect of several enzyme inhibitors on GFP expressing cells. We proposed that inhibiting this GFP fluorescence using small molecule drugs could offer an innovative and effective strategy for evaluating drug penetration in both 2D and 3D models. Thus, our primary objective was to identify universal GFP disruptors.

**FIGURE 1:**
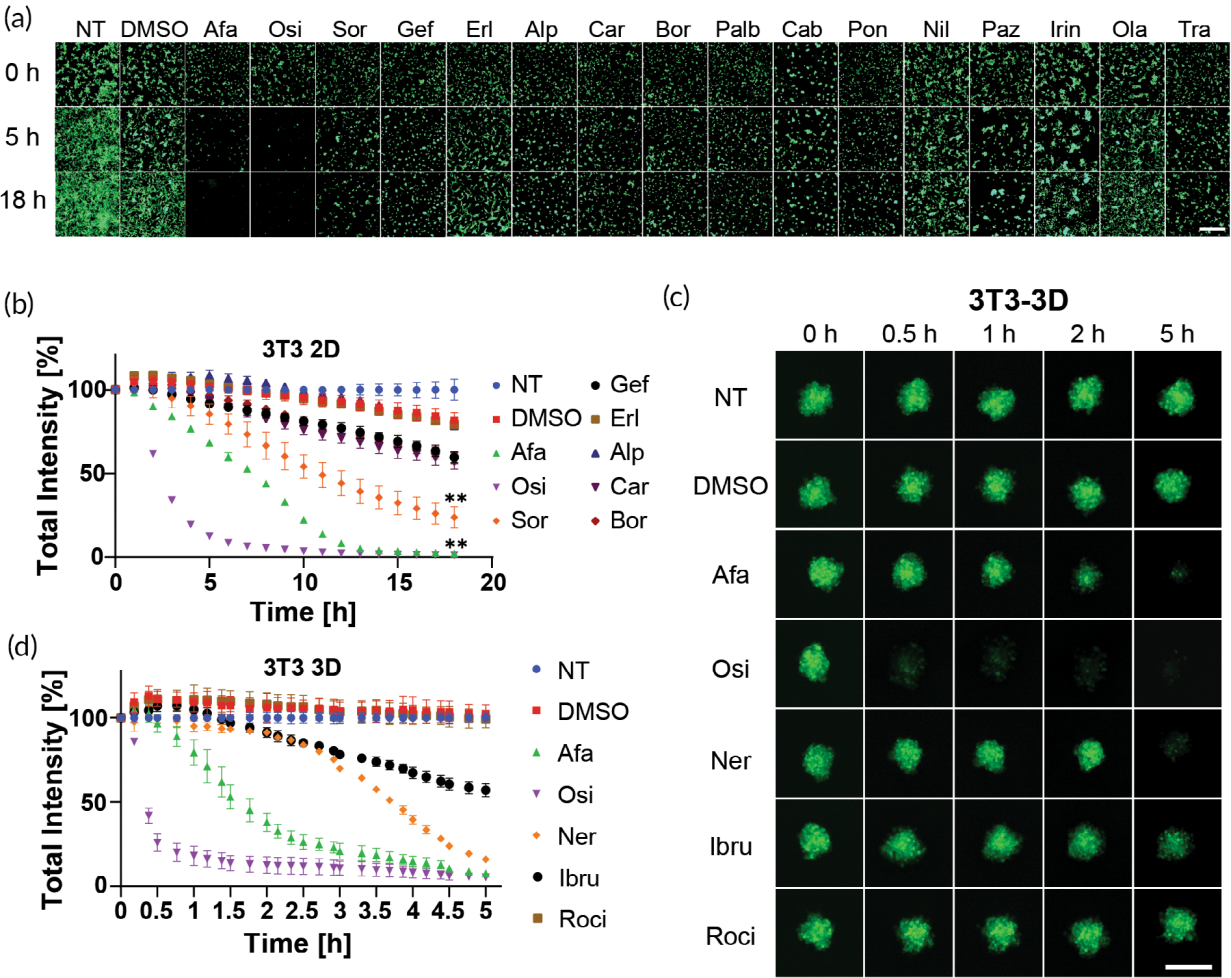
Initial drug screening to discover green fluorescent protein (GFP) disrupting drugs. (a) Representative images taken using LionHeart automated microscope of 3 T3 2D cell culture incubated with various drugs for 18 h, at a concentration of 0.03 mg/mL. Scale bar = 500 μm. (b) Quantification of GFP total intensity signal of 3 T3 2D cell culture in the microscope images, *n* = 2. (c) Representative images taken using LionHeart automated microscope of 24‐h‐old 3 T3 3D cell culture incubated with various drugs, at a concentration of 0.03 mg/mL, for 5 h. Scale bar = 250 μm. (d) Quantification of GFP total intensity signal from the microscope images of 3 T3 3D cell culture incubated with various drugs at a concentration of 0.03 mg/mL for 5 h, *n* = 3. Error bars indicate mean ± SD, **p < 0.01 by unpaired *t*‐test. Green = GFP signal. Non‐treated (NT), afatinib (Afa), osimertinib (Osi), sorafenib (Sor), gefitinib (Gef), erlotinib (Erl), alpelisib (Alp), carfilzomib (Car), bortezomib (Bor), palbociclib (Palb), cabozantinib (Cab), ponatinib (Pon), nilotinib (Nil), pazopanib (Paz), irinotecan (Irin), olaparib (Ola), trametinib (Tra), neratinib (Ner), ibrutinib (Ibru), rociletinib (Roci).

We started with an initial screening of 16 FDA‐approved anticancer drugs, using two concentrations, 0.03 and 0.01 mg/mL, to assess their capability to disrupt GFP signals in 2D cell cultures of 3T3 fibroblasts expressing GFP (ZsGreen) (Figure 1a). Although these are relatively high concentrations, we chose them to increase the possibility of off‐target effects on GFP fluorescence, particularly since many of the drugs we tested were kinase inhibitors. Kinase inhibitors though well tolerated, are known for their broad‐spectrum activity and potential for off‐target effects, with diverse and interesting pharmacology. We monitored and quantified GFP fluorescence over time using automated microscopy and image analysis (see Methods section for detailed protocol).

Analysis of fluorescence intensity of the images identified two drugs, osimertinib and afatinib, hydrophobic small molecules and covalent inhibitors of the EGFR, as the most potent GFP disruptors (Figures 1b and S1a). Drugs with mild disruptive activity included sorafenib, pazopanib, cabozantinib, and trametinib. Osimertinib deactivated GFP significantly faster than the other drugs, although after 18 h both osimertinib and afatinib eventually eliminated GFP fluorescence completely.

Interestingly, the reversible EGFR inhibitors erlotinib and gefitinib did not disrupt GFP emission, suggesting that the covalent inhibitors osimertinib and afatinib likely modify and destabilize GFP. Notably, these inhibitors have an acrylamide moiety, which can bind to cysteine residues.^16^, ^17^ Considering that the GFP protein has two cysteine residues within its beta‐barrel structure,^18^ we hypothesized that disruption of these residues may be responsible for the optical inactivity.

To further evaluate the effects of these drugs in a more physiologically relevant model, we tested osimertinib and afatinib in 3D cell cultures of 3 T3 cells, using the same concentrations as before. In line with our initial findings, both drugs significantly disrupted GFP fluorescent, with osimertinib demonstrating superior potency (Figures 1c,d and S1b,c). Osimertinib reached minimum GFP levels after approximately 100 min, whereas afatinib required around 150 min to achieve similar reductions. Comparative analysis of the effects of these drugs on GFP intensity in 2D and 3D cultures showed that osimertinib had greater potency in the 3D model after 150 min compared to the 2D cell culture (Figure S1d–f).

Next, we expanded our small molecule drug screen to include additional drugs, specifically those with acrylamide moieties and covalent warheads, based on the promising results with osimertinib and afatinib.^16^, ^17^ We continued the drug screening on 3 T3 3D cell culture (Figures 1c and S1g,h). We found that neratinib, another irreversible EGFR inhibitor, displayed potency in disrupting the fluorescence signal, emerging as another potential candidate for real‐time monitoring of drug penetration (Figure 1c,d **)**. Additional drugs that were potential candidates were also kinase inhibitors containing covalent acryl amide, ibrutinib, an irreversible inhibitor of Bruton's tyrosine kinase, and rociletinib, an irreversible EGFR inhibitor which failed clinical trials. Since both showed lower or slower GFP disruptive activity (Figure 1c,d), the three FDA approved covalent EGFR inhibitors, osimertinib, afatinib, and neratinib, were selected for further studies, as they have similar biological targets but display different disruption profiles along with different clinical pharmacokinetic parameters.

### 2.2 Further characterization of GFP disruption of covalent inhibitors

We then conducted tests in two additional cell lines of epithelial cancers, each expressing a distinct fluorescent protein. Cal33 cells expressing enhanced green fluorescent protein (eGFP) from human head and neck squamous cell carcinoma (HNSCC), SK‐136 cells expressing RFP (tdTomato) from murine hepatocellular carcinoma.

**FIGURE 2:**
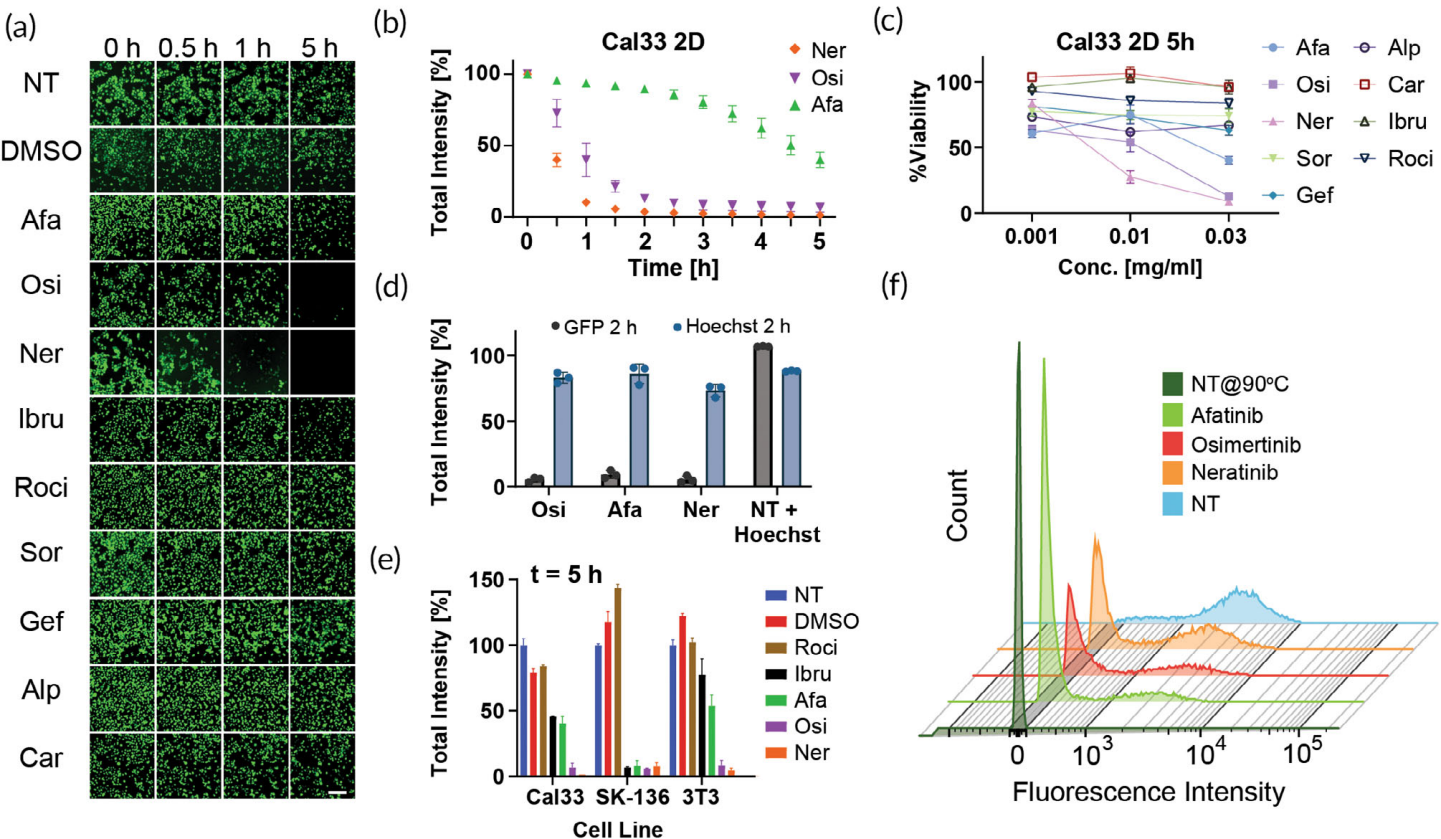
Characterization of green fluorescent protein (GFP) disruption in additional cell lines. (a) Representative GFP images taken using LionHeart automated microscope of Cal33 2D cell culture incubated with various drugs at a concentration of 0.03 mg/mL for 5 h. Scale bar = 250 μm, green = GFP signal. (b) Quantification of GFP total intensity signal from the microscope images of Cal33 cell cultures from the microscope images incubated with afatinib, osimertinib, and neratinib for 5 h, *n* = 3. (c) CTG 2D results of Cal33 cells after 5 h of incubation with various drugs, *n* = 3. (d) Quantification of GFP and Hoechst total intensity signals from the microscope images of Cal33 cell culture stained with Hoechst then incubated with afatinib, osimertinib, and neratinib for 2 h, *n* = 3. (e) Quantification of RFP total intensity signal from the microscope images of SK‐136 2D cell cultures and of GFP total intensity signal of Cal33 and 3 T3 2D cell culture after 5 h of incubation with various drugs, *n* = 3. Error bars indicate mean ± SD. (f) FACS fluorescence intensity histogram of Cal33 24‐h‐old spheroids incubated with 0.01 mg/mL afatinib, osimertinib, and neratinib for 5 h.

In Cal33, we tested nine drugs which included all covalent inhibitors as well as the non‐covalent inhibitor gefitinib, and the covalent proteasome inhibitor carfilzomib. We monitored GFP fluorescence over 5 h with 30‐min intervals (Figures 2a and S2a). We found that neratinib and osimertinib were the fastest and most efficient inhibitors, achieving almost complete inhibition within 2 h, with neratinib showing a significant advantage at the 1‐h mark (Figure 2a,b). We also tested the viability of the Cal33 cells, and the various cell lines in this study, with different drug concentrations to compare drug cytotoxicity. Our results showed a direct correlation between GFP inhibition and cytotoxicity, with a dose‐dependent cytotoxic effect observed at the 5‐h time point (Figures 2c and S3a–d). Although our primary aim was not to directly correlate GFP inhibition with anticancer activity, this finding provides valuable insights. It suggests that the observed GFP inhibition could serve as a potential marker for rapid assessment of drug efficacy and cytotoxicity in these cells, in addition to their permeability evaluation.

To evaluate the effect of the drugs on other forms of fluorophores, we repeated the experiment with nuclear staining and found that there was only a slight reduction in nuclear staining upon incubation with the top three inhibitors (Figures 2d and S2c–e). In addition, we performed a viability assay, where CTG results were normalized to NT cells without Hoechst staining, since a slight decline in viability of the cells that were incubated with Hoechst compared to unstained cells was observed, leading to a decrease in DAPI signal even in untreated stained cells (Figure S2b).

We then compared all irreversible inhibitors in Cal33, SK‐136, and 3T3. Neratinib and osimertinib were the most potent across the different cell lines, regardless of fluorescent protein type (Figure 2e). Ibrutinib only showed potency in SK‐136 2D cell culture; however, the results for 3 T3 2D were less potent than the EGFR inhibitors mentioned before. We observed that the degree of fluorescence disruption varied among the different fluorescent proteins and cell lines. RFP was the most susceptible to disruption, followed by ZsGreen, with eGFP exhibiting the most resistance (Figure S4a–d). Moreover, our analysis indicates a correlation between the CTG results and the observed total intensity values. Notably, at the high concentration of 0.03 mg/mL, all three drugs demonstrated cytotoxicity across all examined cell lines and significantly influenced the decay of GFP fluorescent signals (Figure S3a–d). This differential sensitivity, revealing a hierarchy in the susceptibility of different fluorescent proteins to disruption, could potentially be used in the development of more sophisticated models for drug penetration studies.

Next, we examined the disruption profile of afatinib, osimertinib, and neratinib. While in SK‐136 RFP expressing cells, a distinct difference between the three drugs is not apparent (Figures 2e and S4d,e), in the GFP expressing cell lines (Cal33 and 3 T3), we can observe different profiles. Afatinib has a slow extinguishing profile, and even after 5 h the GFP signal only reaches a minimum of approximately 50% compared with osimertinib and neratinib that show a faster profile that leads to GFP abolishment after approximately 2 h (Figures 2b and S4c,e). Representative images are presented in Figure S4e.

To confirm that these GFP disruptions could be observed in other modalities besides automated fluorescent microscopy, we performed a flow cytometry experiment in 24‐h‐old 3D spheroids of Cal33 after overnight incubation with 0.01 mg/mL of the drugs. We show that GFP inhibition could be easily evaluated in flow cytometry (Figures 2f and S2f–i), and it can be correlated with viability as well (Figure S2f–i). The gate for dead cells was determined by incubating Cal33 untreated cells at 90°C for 5 min. In addition, this process denatured the GFP causing a reduction in the signal's intensity (Figure S2f–h). The results show a GFP signal for neratinib treated cells, indicating that the overnight incubation with 0.01 mg/mL concentration was insufficient to allow full disruption of GFP (Figure 2f).

These results identify the potential of these small molecule drugs with acryl amide warheads to serve as universal disruptors of fluorescent proteins, and their potential application in visualizing and assessing drug penetration in real‐time within 2D and 3D models. Furthermore, the identification of neratinib, osimertinib, and afatinib strengthens the hypothesis that covalent EGFR inhibitors disrupt GFP/RFP via the acrylamide warhead.

### 2.3 Real‐time permeability evaluation in different aged spheroids

Following the successful disruption of GFP and RFP fluorescence in the different 2D cell cultures, we employed Cal33 and SK‐136 spheroids, both of different maturation stages, to test the drugs' permeability as the spheroid matures. Spheroid maturation stages were determined by seeding time, with 24‐, 48‐, 72‐, and 96‐h‐old spheroids representing different stages of spheroid consolidation and extracellular matrix (ECM) formation.

**FIGURE 3:**
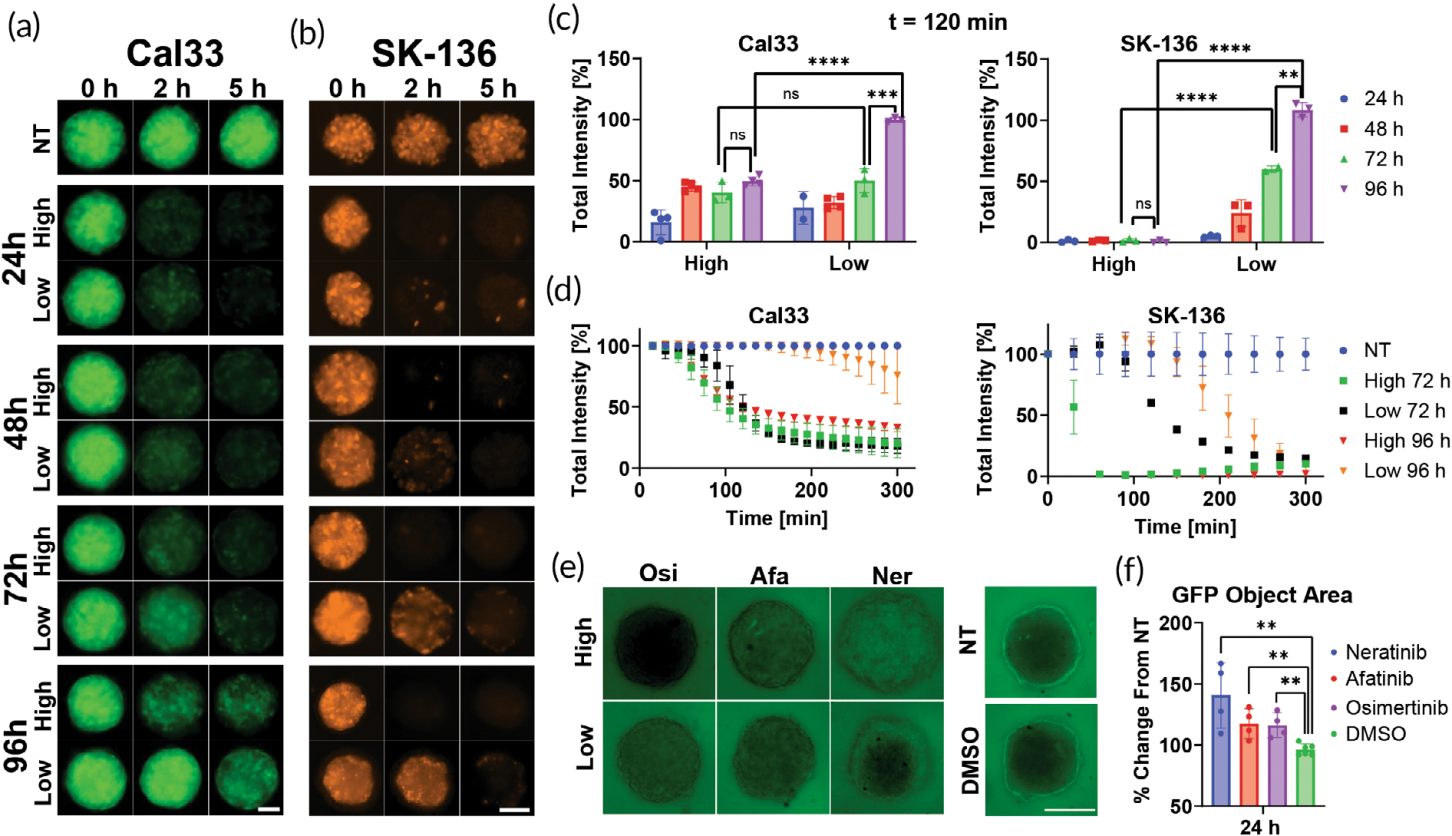
Osimertinib's penetration over time in different aged spheroids and integrity evaluation. (a, b) Representative green fluorescent protein/red fluorescent protein (GFP/RFP) images taken using LionHeart automated microscope of different aged Cal33 (a) and SK‐136 (b) spheroids incubated with two concentrations of osimertinib for 5 h, high = 0.03 mg/mL, low = 0.01 mg/mL. Green = GFP signal, red = RFP signal, scale bar = 100 μm. (c) Quantification of GFP/RFP total intensity signal of different aged Cal33 (left) and SK‐136 (right) spheroids from the microscope images after 2 h of incubation with two concentrations of osimertinib, *n* = 3. (d) Comparison between the GFP/RFP total intensity signal of 72 or 96 h‐old Cal33 (left) and SK‐136 (right) spheroids incubated with two concentrations of osimertinib, for 300 min, *n* = 3. (e) Representative GFP images taken using LionHeart automated microscope of structural integrity assay on 96‐h‐old FaDu spheroids treated with various drugs at a concentration of 0.03 (High) and 0.01 (Low) mg/mL and then incubated with 40 kDa FITC‐dextran for 5 h. Scale bar = 250 μm, green = FITC‐dextran signal. (f) Quantification of the GFP signal inside the spheroids treated with low concentration and incubated with FITC‐dextran for 24 h, using cellular analysis: Object area feature in GEN5+ Data Analysis software, *n* = 3. **p < 0.01, ***p < 0.001, ****p < 0.0001 by unpaired *t*‐test. Error bars indicate mean ± SD.

We treated the spheroids with two different concentrations of osimertinib (Figure 3a,b)—a high dose (0.03 mg/mL) and a low dose (0.01 mg/mL). Interestingly, we found that spheroids aged 24 and 48 h, representing early stages of spheroid formation, exhibited complete permeability to osimertinib at both concentrations (Figure 3c). These results suggest that at these stages, the ECM barriers may not be fully formed or consolidated, thereby allowing the drug to readily penetrate the spheroids.

However, as the spheroids matured (72 h), the rate of drug diffusion was remarkably reduced and appeared to be dose‐dependent. While the high dose of osimertinib diffused rapidly, the lower dose showed a distinctly slower diffusion rate. Even after 5 h, significant fluorescence persisted, particularly in the spheroids treated with the lower drug dose (Figure 3c,d).

A significant reduction in drug permeability was observed in the most mature spheroids of Cal33 (96‐h‐old). Despite treatment, GFP fluorescence remained prevalent, suggesting that the drug was not penetrating the spheroids effectively. A similar effect was observed for SK‐136 96‐h‐old spheroids with the lower drug concentration. This observation is indicative of an increased ECM barrier and more robust cellular junctions as the spheroids mature, effectively limiting the drug's diffusion (Figure 3c,d).

In order to evaluate the effect of the drugs on spheroid integrity, we performed FITC‐dextran penetration assay^19^ on FaDu spheroids (96‐h‐old). In this assay high MW FITC‐dextran does not penetrate a mature spheroid and its penetration can be evaluated with fluorescence microscopy. Upon incubation of the drugs, we noted significant penetration of FITC‐dextran for neratinib at both concentrations and afatinib had a mild but significant effect (*p* < 0.01, Figure 3e,f). We noticed that at high concentration of osimertinib, it seems as some quenching occurred. We hypothesize that this phenomenon is due to optical rather than biological factors, since for the low concentration we received FITC‐dextran penetration similar to that of afatinib, indicating that the integrity of the spheroid was compromised.

These findings demonstrate the inherent complexities of temporal dynamics associated with drug permeability within 3D spheroids. We can monitor the kinetic profile of a complex process which takes into account multiple factors. The differential fluorescence kinetics in spheroids at different maturation stages show how drug permeability can be monitored and evaluated in real‐time. Furthermore, it highlights the critical role of spheroid age, or consolidation stage, in drug permeability—an important consideration for drug development and delivery studies.

### 2.4 Comparison of GFP disruption profiles in three‐dimensional spheroids

**FIGURE 4:**
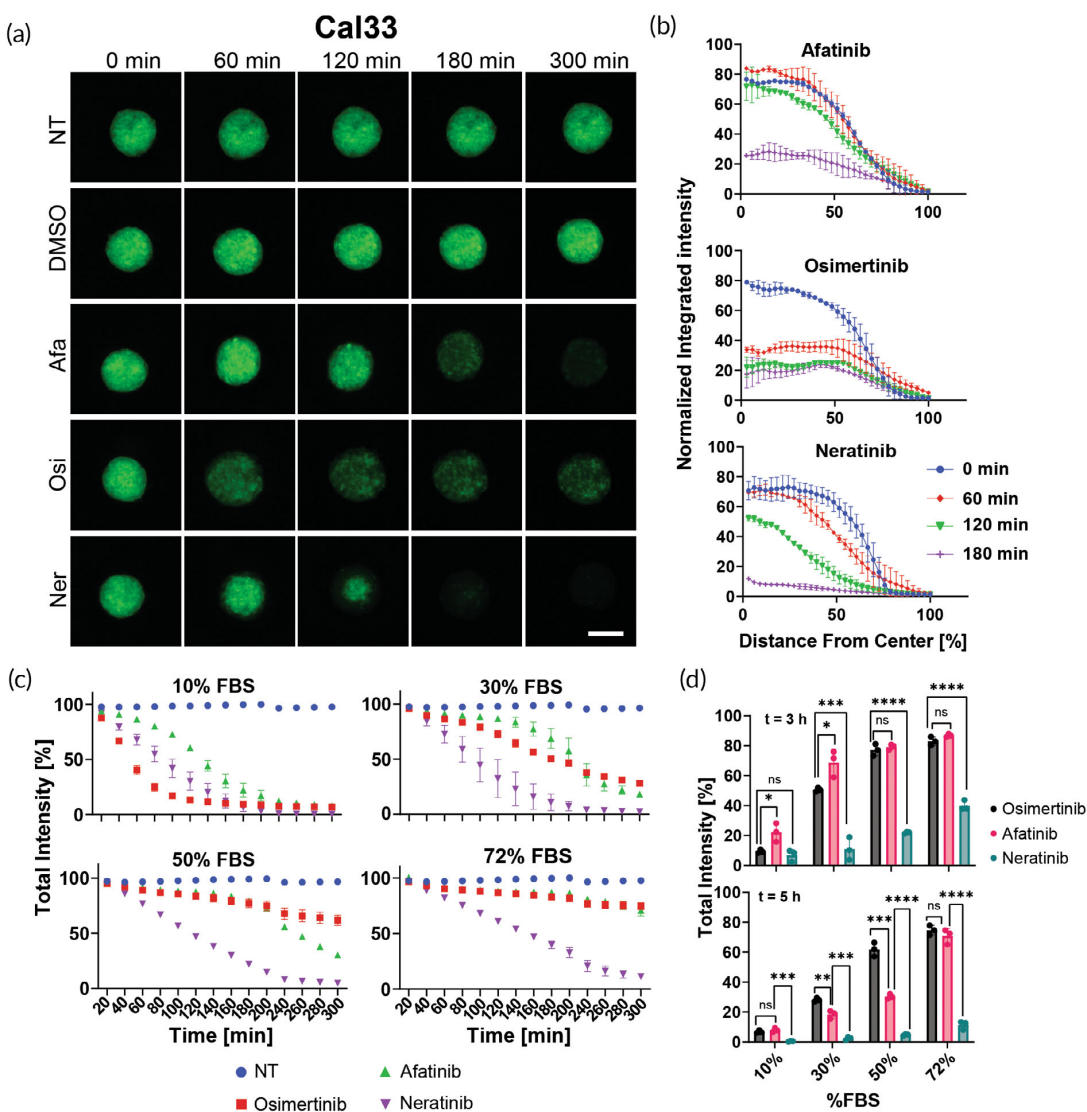
Examining the penetration profile of osimertinib, afatinib, and neratinib in Cal33 3D spheroid model and how different protein concentration modifies it. (a) Representative images taken using LionHeart automated microscope of Cal33 3D spheroids incubated with 0.03 mg/mL of afatinib, osimertinib, and neratinib for 5 h. Green = GFP signal, scale bar = 200 μm. (b) Radial intensity profile at different time points for Cal33 spheroids treated with afatinib (top), osimertinib (middle), and neratinib (bottom), *x* = 0 marks the center of the spheroid, *n* = 2. (c) Quantification of GFP total intensity signal of Cal33 spheroids incubated with afatinib, osimertinib, and neratinib for 5 h at various fetal bovine serum (FBS) concentrations, *n* = 3. (d) Comparison between GFP total intensity signal of Cal33 spheroids incubated with afatinib, osimertinib, and neratinib at *t* = 3 h (top) and 5 h (bottom) at different FBS concentrations, *n* = 3. Error bars indicate mean ± SD. *p < 0.05, **p < 0.01, ***p < 0.001, ****p < 0.0001 by unpaired *t*‐test.

We continued to examine the penetration profiles of different drugs in 3D cell cultures. We chose to focus on 96‐h‐old spheroids based on earlier findings, which revealed that these mature spheroids exhibited the most pronounced variations in osimertinib's penetration profile, depending on its concentration. We incubated 3D spheroids of Cal33 (GFP), SK‐136 (RFP), and K7M2 (GFP + RFP) with various drugs at a concentration of 0.03 mg/mL for 5 h (Figures 4a, S5a, S6a and Supplementary Movies [Link], [Link]). The spheroids were live imaged using automated microscopy and once again, we received that afatinib, osimertinib, and neratinib presented the best GFP/RFP distinguishing abilities after 5 h (Figures 4a, S5a–c,f,g and S6a–c). Ibrutinib was active in SK‐136 but had a less disrupting effect than these three drugs in Cal33 spheroids (Figures 4a and S5a–c). In addition, the images revealed that while osimertinib had a fast penetration profile, it did not achieve complete fluorescence extinguishment. On the other hand, neratinib had a slower penetration profile that follows the pattern of a shrinking ring and reduces fluorescence of GFP/RFP from the outside inside, in what seems to be a controlled fashion. Afatinib has a slower penetration profile than osimertinib and is ultimately less efficient than neratinib (Figures 4a and S5a–c and Supplementary Movies [Link], [Link]). K7M2 is an osteosarcoma (bone cancer) cell line that expresses both mCherry RFP and ZsGreen GFP and we were able to track the fluorescence signal of both proteins (Figure S6b,c). These results demonstrate the same fluorescence signal decay for the tested drugs. In addition, we were able to observe the different effect the drugs have on both the fluorescent proteins, here GFP was more sensitive than RFP.

The trends that were observed in the images were further supported by measuring the normalized integrated intensity throughout the spheroid (see Methods). By focusing on different time points for the same drug, we were able to plot a spatial penetration profile for each drug that is based on the radial distance from the center of the spheorid (Figures 4b and S5e). The plots show significant differences after 60 min. While osimertinib reached a normalized integrated intensity of less than 40 a.u. close to the center of the spheroid (*x* = 0), afatinib and neratinib had an intensity similar to that of the beginning. However, although osimertinib was faster at the beginning, after 120 min the intensity stayed the same, and the minimum remains approximately 20 a.u. at the center. Neratinib, on the other hand, presents a gradual profile until it reaches the lowest value observed out of all three drugs close to the center after 180 min. Similar results for SK‐136 spheroids are presented in Figure S5d.

To ensure the intensity of the GFP/RFP signal remains stable throughout all of our experiments, we compared the GFP intensity of untreated 96‐h‐old Cal33 spheroids from different experiments (Figure S7). These results demonstrate that the GFP total intensity signal remains approximately the same throughout different experiments. Moreover, to minimize the variations caused by different seeding conditions, different passages of the cells and different plates used, our analysis normalized each well to the initial intensity value, therefore eliminating the possible changes in raw values.

We then asked whether the differential penetration profiles of EGFR inhibitors correlate with a key pharmacological parameter that relies on tissue permeability, volume of distribution. Vd is a measure that represents the ability of a drug to leave the bloodstream and penetrate tissues, which is crucial for its therapeutic effectiveness. It is influenced by several factors, including protein binding, tissue permeability, and drug half‐life, making it a complex parameter to model accurately in vitro.^20^

To model Vd, we designed an experiment using four increasing fetal bovine serum (FBS) concentrations in the cell media: 10%, 30%, 50%, and 72%. This approach aimed to account for the drugs' ability to both leave the serum and penetrate the tissue. We measured the total GFP intensity over time (0–300 min) for each FBS concentration, and compared the effects of three covalent EGFR inhibitors (Figure 4c).

In the 10% FBS condition, all three drugs showed rapid and substantial GFP signal reduction, indicating efficient penetration into the spheroids. Osimertinib demonstrated the fastest and most complete signal reduction, followed closely by neratinib, with afatinib showing a slightly slower rate of decline. As serum concentration increased, we observed differential effects on drug penetration. At 30% FBS, osimertinib showed the most significant decrease in inhibition kinetics, followed by afatinib, which also demonstrated reduced efficacy in GFP disruption compared to the 10% condition. Notably, neratinib's performance remained relatively robust. At higher serum concentrations (50% and 72% FBS), the differences between the drugs became more pronounced. Neratinib consistently demonstrated the least sensitivity to increased serum levels, maintaining significant GFP disruption even at 72% FBS (Figure 4c,d). These results demonstrate a clear correlation between GFP disruption kinetics in 3D and the reported clinical Vd values (Table 1). Neratinib's ability to maintain complete efficacy across increasing serum concentrations aligns with its high clinical Vd (6433 L^21^), while osimertinib's greater sensitivity to serum proteins corresponds to its lower Vd (986 L^22^) and long half‐life. Afatinib has been reported to exhibit a high apparent Vd, with values ranging from 1880 to 4500 L,^23^, ^24^, ^25^ with the most evidence pointing to 2700–2800 L. Our data are in agreement with its intermediate Vd value of 2870 L^23^ presenting high correlation in all of the high serum data points (*p* > 0.92) while the 4500 L value is not (*p* = 0.6). Interestingly, the data at 50% serum demonstrated a 0.99 correlation with clinical Vd values (Figure S8).

**TABLE 1:** Pharmacokinetic parameters of the three drugs used in Figure 4.

Drugs	Protein binding (%)	Vd (L)	Half‐life (h)	Relative disruption in 50% serum
Osimertinib	95	986	48–56	1
Afatinib	95	2870	37	3.3
Neratinib	>99	6433	17	10

### 2.5 Evaluating drug permeability in 3D co‐culture spheroid model

In order to investigate the permeability of different tumor spheroids, which do not necessarily express GFP, based on GFP‐disrupting drugs, we established a 3D co‐culture model comprising of GFP‐expressing 3 T3 fibroblasts core surrounded by non‐GFP‐expressing cancer cells. We chose to use fibroblast cells since they are responsible for producing ECM components, such as collagen and proteoglycans.^26^ In addition, it was discovered that cancer associated fibroblasts (CAFs) constitute a vital element within the tumor microenvironment (TME) and exert a significant influence on the advancement of cancer.^27^, ^28^ Incorporating them into a co‐culture spheroid model creates a more realistic cancer spheroid model and provides additional information regarding the permeability of these spheroids. By tracking the GFP‐core fluorescence, we can establish an analysis to quantify the penetration of drugs to the center of the spheroid.

**FIGURE 5:**
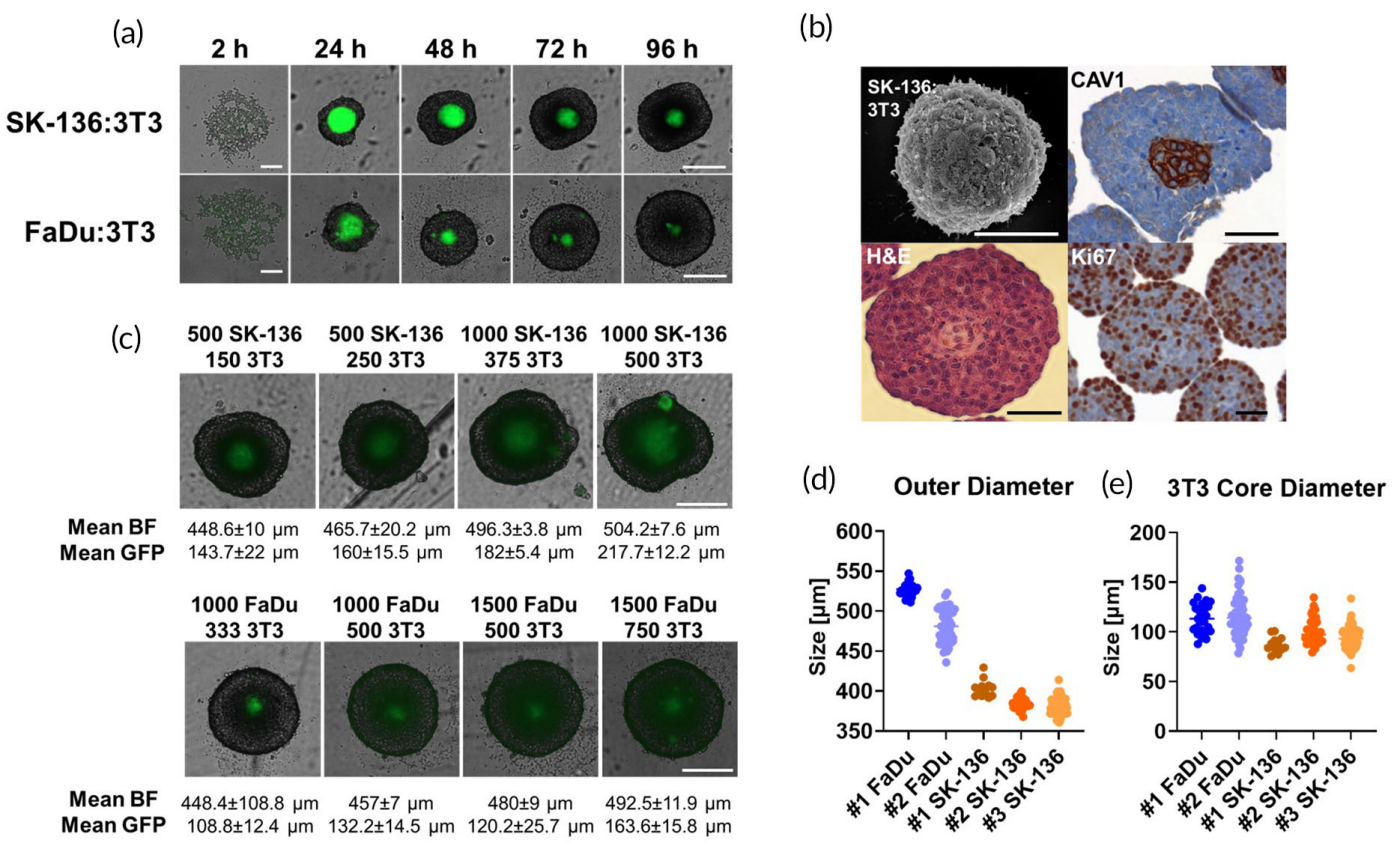
Characterization of co‐culture tumor cells and fibroblasts (3 T3) cells model. (a) Time lapse images of co‐culture tumor: fibroblasts spheroids formation using LionHeart automated microscope with BF and GFP (λ_ex_ = 469 nm, λ_em_ = 525 nm) channels. Three 3 T3 cells in co‐culture with either SK‐136 (upper panel) or FaDu cells (lower panel). Green = GFP signal, scale bar = 250 μm. (b) HR‐SEM images of spheroids of SK‐136:3 T3. CAV1, H&E, and Ki67 staining of SK‐136:3 T3. Scale bars = 50 μm. (c) Representative images of tumor spheroids with different cell amounts and ratios, taken using LionHeart automated microscope with BF and GFP channels. Top = SK‐136:3 T3 spheroids, bottom = FaDu:3 T3 spheroids. Mean BF = spheroid's diameter, mean GFP = green 3 T3 core diameter, scale bars = 250 μm. (d, e) Measurements of outer diameter, *n* > 10 (d) and green core diameter, *n* > 12 (e) from different experiments with same cell seeding amounts; 1000 FaDu:500 3 T3 and 500 SK‐136:250 3 T3. All analyses were performed using Gen5+ image analysis software.

We first conducted a series of characterization experiments to analyze the co‐culture dynamics of tumor cells and GFP‐expressing 3 T3 cells. The cells were seeded in ULA plates to facilitate the formation of co‐spheroids. Using LionHeart automated fluorescence microscope, we captured images of this formation process over a 96‐h period across both Brightfield (BF) and GFP channels. We started with 1000 FaDu cells with 500 3 T3 cells and 500 SK‐136 cells with 250 3 T3 cells per well (Figure 5a). We observe a variation in the GFP signal over time, which we associate with alterations in spheroid density as time progresses, leading to a reduced ability for light to traverse the cellular layers.

We then evaluated the co‐cultured spheroids using scanning electron microscopy (SEM) and histological techniques (Figure 5b and S9a). SEM imaging unveiled the intricate surface morphology of the diverse spheroid types (Figures 5b and S9a). Simultaneously, histological images generated from SK‐136:3 T3 spheroids shed light on the internal cellular organization within these structures. Hematoxylin and eosin (H&E) and caveolin‐1 (CAV1) staining verified the presence of fibroblast cells primarily in the central regions of the spheroids (Figure 5b). Prior research has demonstrated that CAV1 levels rise significantly in confluent fibroblasts,^29^ a trend which we observed as localized staining in areas of cell–cell contact within the fibroblast cells but not within the cancer cells. Ki67 staining highlighted a higher degree of cellular proliferation at the spheroid edges compared to the center, aligning with expectations.

To determine the influence of the fibroblast‐to‐cancer cell ratio on spheroid size and drug permeability, we captured brightfield and GFP images of co‐culture spheroids with various cell ratios (Figure 5c). Gen5+ image analysis software enabled us to measure both the outer diameter of the spheroid (as indicated by mean BF in Figure 5c) and the diameter of the GFP‐expressing 3 T3 core (denoted as mean GFP in Figure 5c). Our analysis revealed a preferable seeding ratio of 2:1 (500 SK‐136 cells: 250 3 T3 cells per well, or 1000 FaDu cells: 500 3 T3 cells per well). This ratio produced spheroids of a round shape with a green core size amenable to image processing. Importantly, this spheroid size permitted an adequate treatment window, with untreated control spheroids maintaining viability. By replicating these cell ratios across all our experiments, we consistently generated spheroids of approximately the same size per plate seeding (Figure S9b,c). This uniformity was further corroborated by measurements of the spheres and the 3 T3 green core diameters in additional replicates (Figure 5d,e).

After establishing the optimal cell ratios of co‐spheroids, we could continue and test the effect of selected drugs on GFP fluorescence in our co‐culture spheroid models. This approach could be an important tool to evaluate drug permeability by measuring how fast the drugs penetrate the spheroids and reach their center.

**FIGURE 6:**
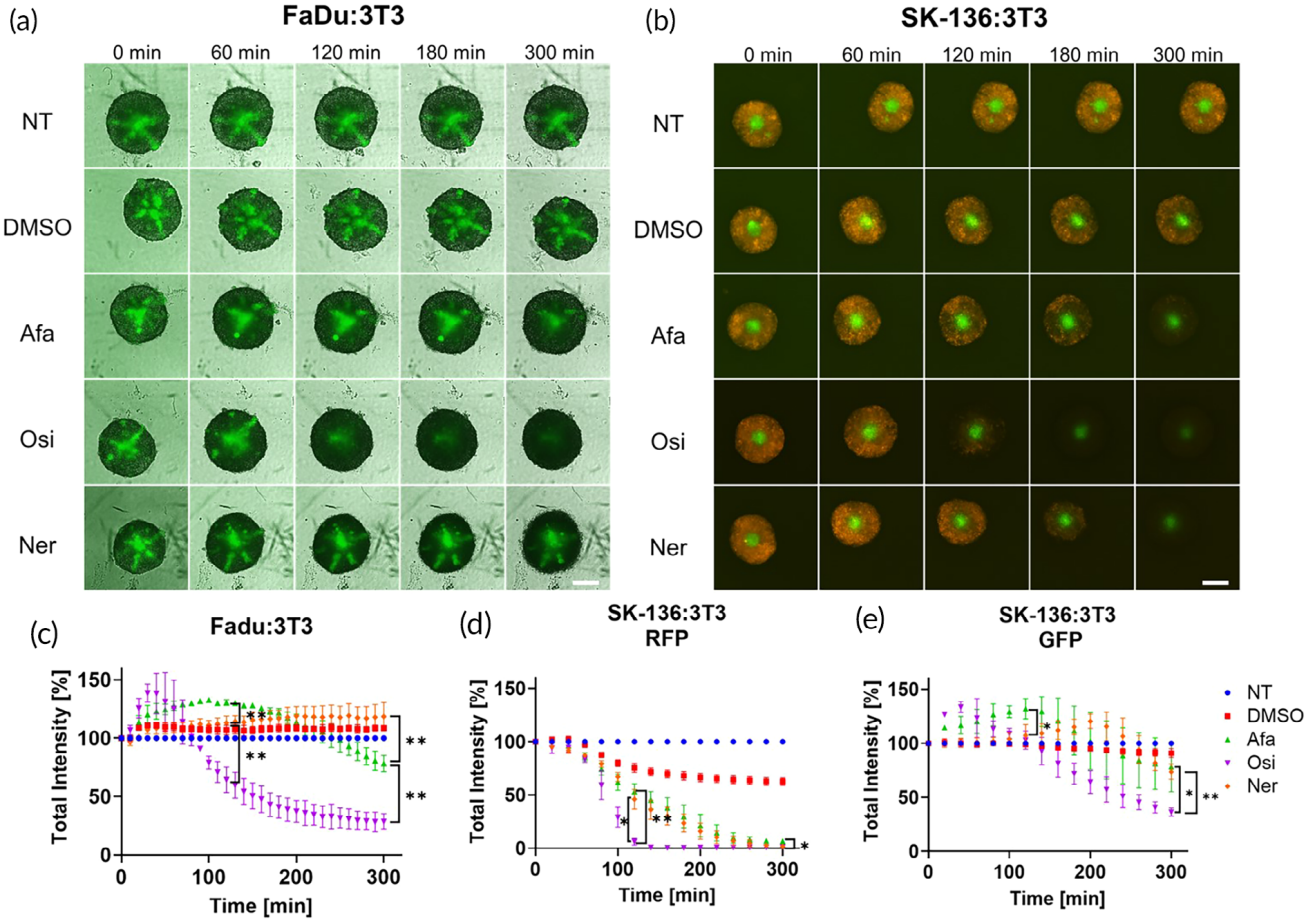
Evaluation of drug penetration via green fluorescent protein (GFP) disruption in 3D tumor spheroid model. (a, b) brightfield and GFP images taken using LionHeart automated microscope of FaDu:3 T3 co‐culture spheroids (a) and GFP and red fluorescent protein (RFP) images or SK‐136:3 T3 co‐culture spheroids (b) incubated with 0.03 mg/mL osimertinib, afatinib, and neratinib for 300 min. Green = GFP signal, red = RFP signal, scale bar = 250 μm. (c) Quantification of GFP total intensity signal of 3 T3 core in FaDu:3 T3 co‐culture, *n* = 3. (d) Quantification of RFP total intensity signal of SK‐136 cells in SK‐136:3 T3 co‐culture spheroids, *n* = 3. (e) GFP total intensity signal of 3 T3 core in SK‐136:3 T3 co‐culture, *n* = 3. *p < 0.05 and **p < 0.01 by unpaired *t*‐test, error bars indicate mean ± SD.

Using automated microscopy, we tracked the co‐culture spheroids of FaDu:3 T3 cells and quantified the GFP intensity over time (Figure 6a,c). Similar to our previous results, we found that osimertinib was superior in disrupting GFP fluorescence in this model compared with afatinib, that was inactive. To our surprise, neratinib was inactive as well. We repeated this experiment in SK‐136:3 T3 co‐culture model and quantified the RFP fluorescence intensity from SK‐136 cell line and GFP fluorescence intensity from 3 T3 cell line (Figure 6b,d,e and Supplementary Movies [Link], [Link]). As demonstrated in previous results (Figures S4 and S5), RFP was more susceptible to disruption and all drugs were able to extinguish the signal, with osimertinib exhibiting the fastest rate of disruption (Figure 6d). However, for the GFP signal we received high permeability for osimertinib, as we saw before, and little to no effect for afatinib and neratinib, unlike the results we received for spheroids of solely SK‐136 cells (Figures 6e and S5a,b).

These results indicate that osimertinib has higher permeability in solid tumors than afatinib and neratinib. Previous studies indicate that even though afatinib is a more potent inhibitor of EGFR than osimertinib (for WT EGFR, IC50 of 0.5 nM for afatinib^30^ compared to 493.8 nM for osimertinib^31^), osimertinib had an advantage in clinical trials of non‐small cell lung cancer (NSCLC) with brain or central nervous system (CNS) metastases.^32^, ^33^ This may be explained by its superior permeability in solid tumors, as demonstrated in our study.

### 2.6 Exploring controlled release with dual‐drug nanoparticles

**FIGURE 7:**
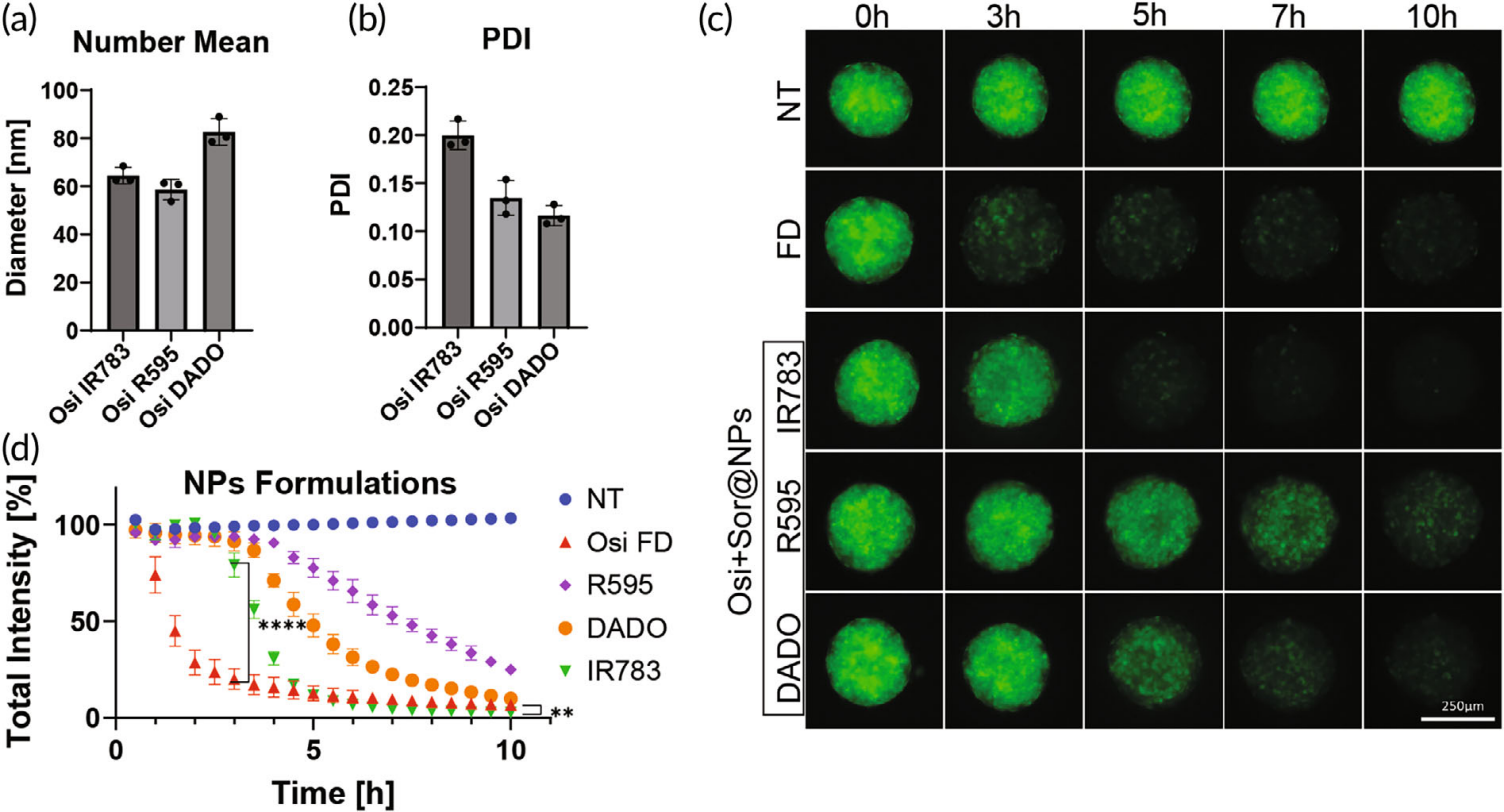
Controlled release using dual‐drug nanoparticles. (a, b) dynamic light scattering (DLS) measurements of the different nanoparticles diameter (a) and poly dispersity index (PDI) (b), *n* = 3. (c) Representative green fluorescent protein (GFP) images taken using LionHeart automated microscope of Cal33 spheroids incubated for 10 h with 0.03 mg/mL of osimertinib free drug (FD) and nanoparticles (NPs) of osimertinib with sorafenib with different stabilizers, green = GFP signal. (d) Quantification of GFP total intensity signal of dual‐drug nanoparticles compared with osimertinib free drug, *n* = 4. **p < 0.01, ***p < 0.001, ****p < 0.0001 by unpaired *t*‐test. Error bars indicate mean ± SD.

We continued by testing the effect that nano formulations have on the kinetic profile of GFP/RFP disruption. The nanoparticles were prepared based on a previously reported method that facilitates the nanoprecipitation of drug nanoparticles, with IR783 serving as a stabilizing agent.^34^ This method was further developed to include dual‐drug encapsulation and additional stabilizers.^35^ In this work, different classifications are used for small molecule drugs to predict their ability to form stable self‐assembled nanoparticles. According to the reported model, osimertinib is predicted to be a drug that will not form stable nanoparticles on its own. In order to prepare stable nanoparticles, we had to combine it with a stabilizing drug (see Methods). We chose sorafenib, a drug that has shown no GFP shutdown effect in our initial screen on 3 T3 2D cell line and that was found to be an efficient co‐drug stabilizer. In addition to IR783, we used two more stabilizing agents, R595^36^ and PDA‐PDO‐In820 (DADO)^37^ and examined their penetration profiles. The three types of nanoparticles were prepared and characterized for their size and poly dispersity index (PDI) by dynamic light scattering (DLS) and drug content by HPLC (Figures 7a,b and S10a–c). The nanoparticles and free drugs were incubated with Cal33 96‐h‐old spheroids for 10 h, at a concentration equivalent to that of the free drug we tested previously, 0.03 mg/mL. We compared the GFP fluorescence total intensity signal of osimertinib as free drug (FD) and as dual‐drug nanoparticles with the different stabilizers, and with sorafenib nanoparticles with the different stabilizers as control (Figures 7c,d and S10d). Osimertinib and sorafenib nanoparticles exhibited slow release, it took approximately 2 h until an initial disruption could be observed. After 3.5 h we receive a significant decrease to approximately 60% for IR783 nanoparticles, followed by DADO nanoparticles at 4.5 h. R595 nanoparticles only reach this value at 6.5 h and even after 10 h, the GFP signal has reached only 25%. The penetration profile was similar for both free drug and nanoparticles, showing slow decline over time. Only IR783 nanoparticles were able to reach full disruption similar to that of osimertinib as free drug. Moreover, we examined whether osimertinib and sorafenib present a synergistic effect using Zip analysis method (Figure S10e). We received a synergy score of 4.23 indicating an additive interaction between the drugs.

By comparing different nanoparticles of the same drug at equivalent doses, we can attribute the stability and penetration kinetic profile of different formulations, inducing either controlled release or improved penetration.

### 2.7 Comparison of penetration enhancers in 3D spheroids

Next, we sought to test if we can enhance drug diffusion into tumor spheroids with penetration enhancers. Though most permeability and penetration enhancers are commonly used to increase oral bioavailability of drugs and macromolecules,^38^ we thought to explore whether penetration enhancers can be used in solid tumors as well.

We first looked at several penetration enhancer compounds such as methanol, ethanol, beta‐cyclodextrin (β‐CD), and trypsin‐Ethylenediaminetetraacetic acid (EDTA). Ethanol and methanol serve as permeation enhancers for transdermal drug delivery. Ethanol enhances the permeation of polar and nonpolar molecules and methanol improves the transdermal transport of hydrophilic and lipophilic drugs.^39^, ^40^, ^41^, ^42^ Cyclodextrin can improve the permeability of insoluble drugs through the membranes.^43^, ^44^, ^45^ Trypsin is a proteolytic enzyme that was shown to enhance the permeation of drugs, including protein drugs, and macromolecules through membranes.^46^, ^47^, ^48^ SNAC (sodium N‐[8‐(2‐hydroxybenzoyl)amino]caprylate) is a novel absorption enhancer that promotes the oral bioavailability of poorly permeable drugs by facilitating their transport across the intestinal epithelium, primarily through transcellular pathways, and has gained attention for its potential to enable oral delivery of peptides and other macromolecules that are typically administered parenterally.^49^ We performed preliminary experiments to determine the concentrations of the compounds that would not damage the GFP‐signal.

**FIGURE 8:**
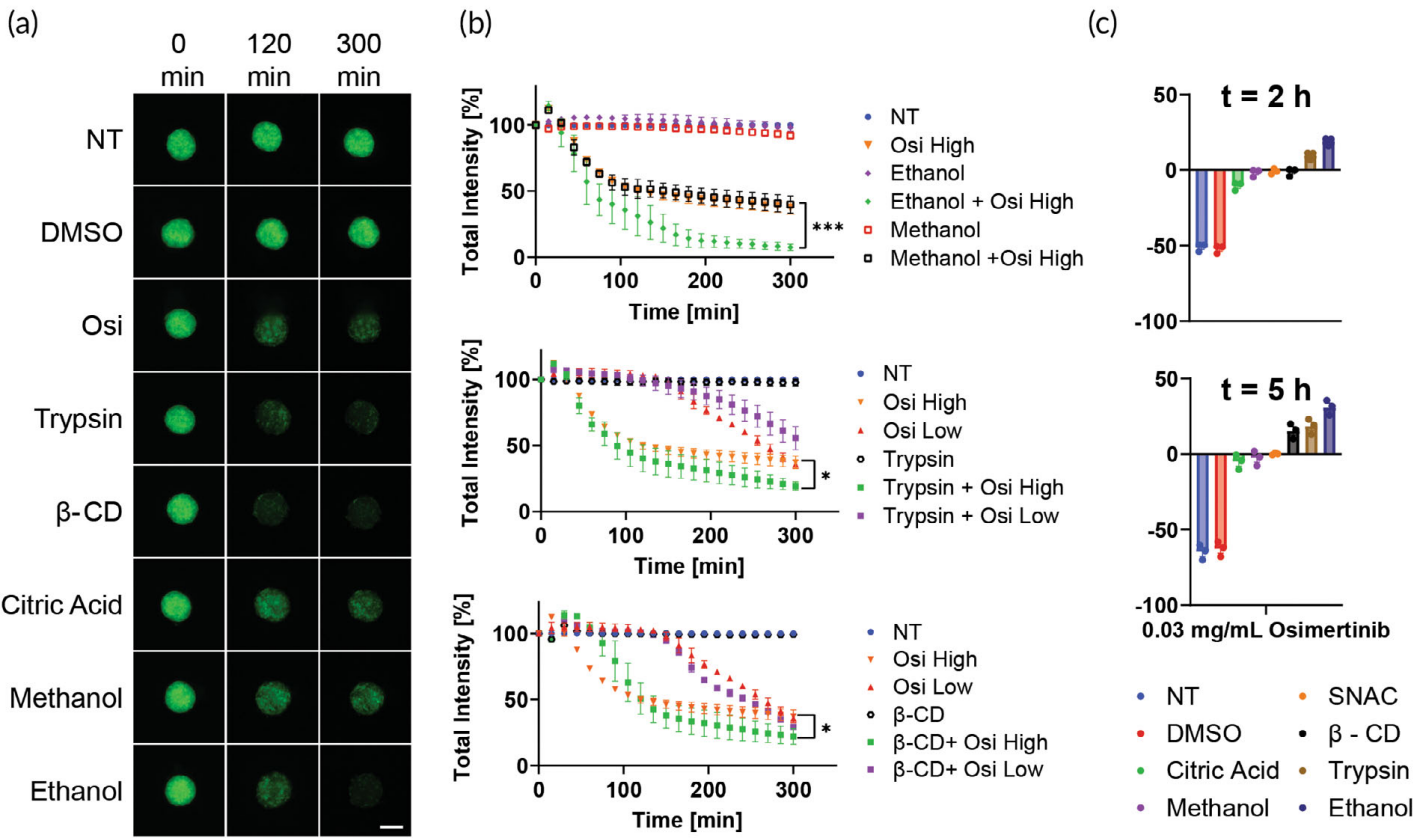
Penetration enhancement assay. (a) Representative green fluorescent protein (GFP) images taken using LionHeart automated microscope of 96‐h‐old Cal33 spheroids incubated with various compounds and with 0.03 mg/mL of osimertinib for 5 h. Green = GFP signal, scale bar = 200 μm. (b) Quantification of GFP total intensity signal of Cal33 spheroids incubated with different concentrations of osimertinib (high = 0.03 mg/mL, low = 0.01 mg/mL) together with ethanol/methanol (top) trypsin–EDTA (middle) and β‐CD (bottom), *n* = 3. (c) Quantification of the relative percentage of improvement each compound had compared to osimertinib alone after 2 h (top) and 5 h (bottom) of incubation, *n* = 3. ***p < 0.001, **p < 0.01, *p < 0.05 by unpaired *t*‐test, error bars indicate mean ± SD.

We used matured 96‐h‐old Cal33 spheroids which showed reduced permeability for osimertinib low dose (0.01 mg/mL) and presented incomplete efficacy at 5 h. Spheroids were incubated with the various compounds for 2 h, followed by the addition of osimertinib at high and low doses and monitored with automated fluorescence microscopy for 5 h (Figure 8a). The images were analyzed and the relative percent of improved penetration from osimertinib as free drug was quantified, allowing us to rank the enhancers based on their effectiveness in these settings (Figure 8b,c). We observed that there are stark differences between the different compounds with some unexpected results. Surprisingly, ethanol significantly enhanced penetration while methanol had no effect at the same concentration. (Figure 8b). β‐CD presented a significant improvement in penetration in both low and high concentrations of osimertinib while trypsin only improved the high concentration (Figure 8b,c). SNAC and citric acid has no significant effect at any concentration.

We concluded that real‐time monitoring of GFP disruption can indeed be used to evaluate compounds which influence the permeability of spheroids. By finding the right combination of enhancer and drug, it may be possible to improve the treatment of solid tumors in cancer therapy.

## 3 DISCUSSION

Drug permeability in solid tumors is a major challenge for cancer therapy, as effective drug distribution is crucial for therapeutic success. While 3D models represent a significant step toward modeling drug penetration, they still lack the ability to provide continuous, real‐time data on drug behavior within the tumor matrix. This limitation is largely due to the impracticality of current imaging methods, such as fluorescence imaging, which, despite their potential, are hindered by the tediousness of sample preparation, susceptibility to photobleaching, and slow data acquisition.

Fluorescent proteins are major tools in biomedicine, extensively used for imaging and tracking cellular processes for decades. Despite its widespread application, to date, there is no selective low MW inhibitor specifically inhibiting GFP signal irreversibly. In addition, it is important to note that GFP signal in living cells does not necessarily relate immediately to their viability. GFP signal can persist sometime after cell death or decrease during cell viability.

In this study, we screened for drugs which demonstrated the most efficient disruption to GFP or RFP fluorescence intensity across multiple cell lines. Since no commonly known GFP or RFP disruptors exist, we screened mainly kinase inhibitors which have in several cases off‐target effects, and are well tolerated. All leading candidates discovered in our 2D screen were covalent EGFR inhibitors with acrylamide warheads. Since reversible EGFR inhibitors did not display the same effects, they presumably disrupt cysteine residues required for fluorescence. Though we also identified two other candidates, ibrutinib and rociletinib, in this study we focused on osimertinib, afatinib, and neratinib which were more potent and demonstrated different disruption profiles in 3D spheroids.

Once we identified the lead candidates, we tested their kinetics in different settings in order to correlate drug penetration in different in vitro tumor models. We have demonstrated that these drugs can assess the permeability of different spheroids, delivery systems, and permeability enhancers. Importantly, we found that the differential kinetics in 3D spheroids correlated perfectly with their known clinical volume of distribution (Vd) properties when high serum concentrations were applied. This correlation suggests that our assay captures meaningful differences in drug behavior that relate to their clinical pharmacokinetic properties. The relevance of our GFP readout to permeability was demonstrated through multiple experimental approaches, including high serum and Vd correlation, nanoparticle formulations, and the application of penetration enhancers like cyclodextrin and trypsin‐EDTA.

For instances that the tumors are not expressing GFP, we developed a model where we trapped GFP‐expressing cells in the center of spheroids to compare the rate of fluorescent decay, and found that liver cancer cells (SK‐136) were less permeable than head and neck cancer cells (FaDu). Additionally, our comparative testing showed that ethanol was the most efficient permeability enhancer, while methanol was surprisingly inefficient.

Current assays for determining spatial cell viability in 3D, such as the Live/Dead assay, typically involve the use of fluorescent dyes that stain living cells differently from dead cells, providing a clear distinction based on membrane integrity and metabolic activity. These assays, while effective, often require multiple staining and washing steps, which can be labor‐intensive and time‐consuming, and may not offer continuous real‐time monitoring of cell viability. While Live/Dead assays are indeed widely used and provide valuable spatial information on drug activity, we believe that monitoring GFP disruption offers several distinct advantages:

1. Speed and Efficiency: Monitoring GFP disruption allows for real‐time assessment of drug penetration and effects without the need for additional staining or washing steps, which are required in Live/Dead assays. This makes the process quicker and less labor‐intensive.
2. Cost‐Effectiveness: GFP disruption assays are more cost‐effective as they do not require the purchase of specialized kits or reagents needed for Live/Dead assays. This makes them accessible for high‐throughput screening.
3. Simplicity and High‐Throughput: The GFP assay can be easily integrated into high‐throughput screening platforms, facilitating the rapid evaluation of multiple compounds simultaneously. This is particularly advantageous for large‐scale drug screening efforts.
4. Noninvasive and Continuous Monitoring: GFP disruption can be monitored continuously over time, providing dynamic insights into the kinetics of drug penetration and action. This noninvasive approach allows for longitudinal studies without perturbing the spheroid environment.
5. Versatility: Our GFP‐based approach can be easily adapted to different experimental setups and does not require specific modifications to accommodate various drug types or formulations.

### 3.1 Study limitations

We acknowledge the complexity and potential limitations of our approach. One major limitation is that GFP/RFP disruption is an off‐target effect of the EGFR inhibitors, necessitating relatively high drug concentrations (0.01–0.03 mg/mL) to achieve the desired results. This concentration aligns with the peak plasma concentrations (Cmax) achieved by several widely used kinase inhibitors in clinical settings, such as sorafenib and pazopanib, which reach Cmax values within the range of 10.9–132 μM. While these concentrations are useful for testing pharmacokinetic hypotheses, future studies should focus on developing drugs with more potent GFP inhibition at lower concentrations.

The relationship between fluorescence intensity decrease and drug penetration is a simplified representation of the complex processes occurring within spheroids. Factors such as cytotoxicity, cell death, and cellular stress‐related signaling cascades could also influence the observed fluorescence intensity decline. Despite this, we observed distinct kinetic profiles for different drugs in our 3D model, which cannot be solely attributed to general cytotoxicity or cellular stress responses.

Additionally, we recognize the need for validation in a broader range of cancer models and normal cells to ensure the generalizability of our results. Although our study included various cell lines, further validation across different cancer types and more extensive sample sizes would strengthen the applicability of our findings.

The irreversible disruption of GFP/RFP fluorescence by osimertinib, afatinib, and neratinib serves as a novel readout for drug penetration and efficacy in our 3D spheroid model. While the exact molecular mechanism remains to be fully elucidated, our data suggest two potential mechanisms for this disruption: direct interaction between the acrylamide warhead of these inhibitors and cysteine residues within GFP/RFP, and an indirect effect tied to cytotoxicity, indicating cell stress and integrity loss. These findings highlight that the observed disruption correlates not only with drug penetration but also with drug efficacy and distribution. A deeper understanding of these disruption mechanisms could reveal how specific interactions between the drugs and the fluorescent proteins lead to fluorescence disruption. This knowledge would enable us to design small molecules with enhanced potency and selectivity for GFP/RFP inhibition. By improving the potency of these molecules, it would be possible to achieve fluorescence disruption without cytotoxicity. Exploring this phenomenon with newly designed small molecules could pave the way for a highly efficient research tool in pharmacology and drug delivery. These advanced inhibitors could serve dual functions: as therapeutic agents targeting cancer cells and as tools for in situ tracking of drug distribution. This dual functionality would allow researchers to simultaneously monitor drug penetration and efficacy in real‐time, providing critical insights into the behavior of drugs within the tumor microenvironment. Such advancements could optimize therapeutic strategies for solid tumors, particularly those with low permeability. By precisely tracking drug distribution and understanding the factors that influence drug penetration, researchers could develop more effective treatment regimens. Ultimately, this approach could lead to more personalized and effective cancer therapies, improving patient outcomes and advancing the field of cancer pharmacology.

## 4 CONCLUSIONS

In summary, we found that covalent EGFR inhibitors have an off‐target effect that enables them to disrupt GFP/RFP fluorescence. While the off‐target disruption presents certain limitations, it also uncovers an innovative method for studying drug distribution and efficacy in solid tumors. The potential applications of this approach are vast, ranging from the development of more effective cancer treatments to insights into tumor heterogeneity and drug resistance. Monitoring GFP disruption offers several advantages over traditional methods, including speed, efficiency, cost‐effectiveness, simplicity, and the ability to provide continuous, real‐time data. With further refinement and understanding of the underlying mechanisms, this methodology could become a cornerstone in the advancement of cancer pharmacology and personalized medicine.

## 5 MATERIALS AND METHODS

### 5.1 Materials and reagents

All nondrug chemicals were purchased from Sigma Aldrich (St. Louis, MO, USA). DMSO was purchased from Carlo Erba (Emmendingen, Germany), sodium bicarbonate was purchased from Bio Lab Chemicals (Jerusalem, Israel). All drugs were purchased from LC‐Laboratories (Woburn, MA, USA) and MedChemExpress LLC (NJ, USA).

### 5.2 Cell cultures

The SK‐136‐RFP (tdTomato) cell line was generated and harvested from c‐Myc and β‐catenin amplified hepatoblastoma cells from FVB mice in the lab of Daniel Heller, Memorial Sloan Kettering Cancer Center, USA, which kindly shared them with us.^50^ 3 T3‐ZsGreen expressing fibroblasts cell line, was kindly shared with us from Daniel Heller's lab as well.

Cal33 expressing eGFP and FaDu cell lines were a kind gift provided by the lab of Moshe Elkabets, Ben‐Gurion University, Israel.

K7M2 (mCherry RFP and ZsGreen GFP) is an osteosarcoma cell line that was a kind gift from Yuval Shaked lab, Technion, Israel.

All cell lines were cultured in Dulbecco's Modified Eagle Medium (DMEM) supplemented with 10% FBS, 2 mM L‐glutamine, Penicillin G Sodium Salt: 100 units/mL and Streptomycin Sulfate: 0.1 mg/mL (all purchased from Biological Industries, Israel). All cells were incubated at 37°C with 5% CO_2_ and 65% humidity.

### 5.3 Drug permeability evaluation via green fluorescent protein/red fluorescent protein disruption assay

Two‐dimensional experiments: 3 T3, Cal33, or SK‐136 cells were seeded in 96 well plates (6000, 10,000, and 6000 cells per well accordingly), and were allowed 24 h to adhere. The following day, drugs dissolved in DMSO and diluted in DMEM to a concentration of 0.03 and 0.01 mg/mL were added to the wells for 18 h, untreated cells were used as control. For SK‐136 the medium was replaced with Hanks' balanced salt solution (HBSS) with 10% FBS and the drugs were diluted with HBSS as well to reduce auto fluorescence caused by phenol red in the DMEM medium.

Three‐dimensional experiments: Approximately 250, 500, 2000, 1000 cells of 3 T3, SK‐136, Cal33, and K7M2 (accordingly) were seeded on ultra‐low attachment (ULA) round‐bottom 96 well plates (Corning, USA) to create 3D tumor models and were allowed 96 h to form a sphere. For 3 T3, the spheres were 24‐h‐old.

To generate co‐culture spheroids, approximately 500 SK‐136 cells or 1000 FaDu cells were seeded at a ratio of 2:1 with 3 T3 cells on ULA round‐bottom 96 well plates. The spheroids were allowed to grow for 96 h. After 96 h, drugs dissolved in DMSO or nanoparticles (NPs) dissolved in DDW were diluted in DMEM to a concentration of 0.03 mg/mL and were added to the wells for 5 h, untreated spheroids were used as control.

### 5.4 Cell viability assay

The viability of both 2D and 3D cell cultures was quantified using Promega® (Madison, WI, USA) CellTiter‐Glo® (CTG) 2D or 3D kit, according to the manufacturer's instructions.

### 5.5 Nuclear staining assay

Cal33 cells in 2D culture were incubated with 0.005 mg/mL of Hoechst (33342, Invitrogen, Thermo Fisher Scientific, Waltham, MA) for 20 min, then washed with fresh medium and 0.03 mg/mL of drugs were added for 5 h incubation. We imaged the cells using LionHeart automated microscope with three channels—brightfield, GFP (λ_ex_ = 469 nm, λ_em_ = 525 nm), and DAPI (λ_ex_ = 377, λ_em_ = 447).

### 5.6 Structural integrity assay

FaDu spheroids (96‐h‐old) were incubated with 0.03 and 0.01 mg/mL drugs for 5 h. After 5 h 17.44 mg/mL FITC‐dextran (40 kDa, sulfate and sodium salt, catalog number 78331, Merck) were added to each well and images were taken after 5 and 24 h of incubation. To determine the GFP signal of the FITC‐dextran inside the spheroid, we used GEN5+ Data Analysis software (BioTek, Agilent Technologies, Santa Clara, CA, USA) “Cellular analysis: Object area” feature, where we defined a primary mask based on BF images and quantified the GFP signal inside the mask. We then normalized the value to NT spheroids.

### 5.7 Flow cytometry assay

Experiments were conducted using BD LSR‐II analyzer flow cytometer with excitation at 488 nm and emission filter of 530/30 nm.

Initial calibration and gate setting: 4 × 10^6^ non‐treated Cal33 cells were divided into two Eppendorf tubes, one was incubated at 90°C for 5 min to determine the gate for dead cells while the other was used as live control. Gates were set as P1‐live and P2‐dead using Forward Scatter‐A/Side Scatter‐A dot plots. In addition, we used 530/30 filter for count versus Intensity histograms.

Single cell viability assay: 2 × 10^6^ Cal33 cells in Eppendorf tube were incubated with 0.03 mg/mL of afatinib for 10 min or 5 h.

3D assay: 1 × 10^6^ Cal33 cells were seeded into ULA Flasks and allowed 24 h to form spheroids. Subsequently, drugs were added at a concentration of 0.01 mg/mL and after overnight incubation the spheroids were transferred to 15 mL Falcon tubes and centrifuged at 4010 rpm for 4 min. The supernatant was discarded, and 1 mL of trypsin was added to the spheroids, followed by gentle pipetting and a 10‐min incubation. Then, 5 mL of medium was added, and the spheroids were aggressively pipetted to dissociate them into single cell and an additional centrifugation was performed to concentrate the sample.

### 5.8 High serum assay

A total of 2000 Cal33 cells per well were allowed 96 h to form spheroids. FBS concentrations were adjusted by removing DMEM from the wells and adding FBS and DMEM only, to a final volume of 250 μL in each well according to desired FBS concentration (10%, 30%, 50%, and 72%). Non‐treated wells had the same FBS concentration as the relevant treated wells. Osimertinib, afatinib, and neratinib were added to treated well at a concentration of 0.03 mg/mL and were live imaged for 5 h.

### 5.9 Enhancing permeability assay

Reagents were added to 96‐h‐old Cal33 spheroids at varying concentrations, one treatment per well; 35 μL trypsin‐EDTA solution (0.25%, Biological Industries, Beit‐Haemek, Israel), 22 μL of ethanol (96%) or methanol, 0.13 mg/mL of methyl‐beta‐cyclodextrin (Holland Moran, catalog number 377110050), 0.2 mg/mL of citric acid monohydrate, and 0.2 mg/mL of salcaprozate sodium (SNAC) (Tzamal, catalog number CS‐0081977). Osimertinib was then added at two concentrations: 0.03 and 0.01 mg/mL and its permeation ability was evaluated using automated microscopy for 5 h.

### 5.10 Imaging and image analysis

Images were acquired using LionHeart FX automated fluorescence microscope (BioTek, Agilent Technologies, Santa Clara, CA, USA) with different channels: Brightfield (4X PL ACH), GFP (λ_ex_ = 469 nm, λ_em_ = 525 nm), and RFP (λ_ex_ = 531, λ_em_ = 593). All imaging experiments in this work were performed on live cells and spheroids. Images were preprocessed and analyzed using the supplier image analysis software, Gen5+ Data Analysis. To quantify the fluorescence intensity, “image statistics‐ total intensity analysis” tool was used with adjusted threshold per experiment. Minimum thresholds were determined for every experiment depending on the background of the images.

Size measurement: The spheroids' outer diameter and core size were measured using cellular analysis tool. For outer diameter the main channel was brightfield, with a threshold value of 17,000, minimum size of 100 μm for SK‐136, 200 μm for Cal33, and 300 μm for FaDu. A rolling ball diameter of 700 μm, and image smoothing strength of 20 cycles of 3 × 3 average filter, was applied for preprocessing. Core size was measured using a similar analysis for the GFP channel with a threshold value of 4000 and a minimum size of 50 μm.

Permeability evaluation: Analysis for drugs' permeability was performed using a radial intensity profiler FIJI (ImageJ) plugin^51^ applied on images acquired with LionHeart automated microscope with GFP channel. This plugin measures the total fluorescence intensity around concentric circles and produces a profile plot of these values as a function of the radial distance from the center. The intensity at a specific distance from the center point is obtained by summing up the pixel values around a circle of that radius. The integrated intensity is then divided by the number of pixels within the circle, resulting in comparable normalized values. In order to compare different sized spheroids, the same sized mask was used for all spheroids and the radial distance was normalized by dividing the measurement of each spheroid by the maximum radius measured.

### 5.11 Preparation of nanoparticles

Approximately 100 μL of drugs dissolved in DMSO (concentration of 10 mg/mL, 1:1 ratio) were added under slight vortex to an aqueous dye solution containing 100 μL of various stabilizers (IR783 2 mg/mL,^34^ R595^36^ 10 mg/mL, and PDA‐PDO‐In820^37^ 2 mg/mL) buffered with 500 μL of 0.1 M sodium bicarbonate. Centrifugation was used for purification (30,000 rpm, 15 min, 25°C) and the pellet was resuspended in 1 mL DDW. The solution was sonicated using Sonics' Vibra‐cell ultrasonic processor (20% amplitude, 3 s pulses) until it became homogeneous.

### 5.12 Characterization of nanoparticles

Size: Dynamic light scattering measurements were conducted using a Zetasizer Nano ZS (Malvern Panalytical, Malvern, UK). For the measurements, the samples were diluted 10× with DDW. The particles size as hydrodynamic diameter was calculated from the size distribution by intensity. The results are reported as the average of three independent measurements ± the deviation from the mean. The uniformity of size distribution was recorded as Poly Dispersity Index (PDI) obtained with the particles size.

Drug loading: NPs diluted 1:10 in acetonitrile solution were measured with ultra‐performance liquid chromatography (Acquity arc UPLC, Waters Corp, MA) using a CORTECS C18 (4.6 × 50 mm 2.7 μm) column.

### 5.13 Drug synergy evaluation

Synergy evaluation was calculated using the SynergyFinderPlus tool (Netphar, Faculty of Medicine, University of Helsinki, Finland) with the Zero Interaction Potency (ZIP) method. This method predicts the expected effect of two drugs under the assumption that they do not interact with each other and presents a synergy score. A ZIP score greater than 10 indicates the interaction between the two drugs is likely synergistic, results between −10 and 10 suggest an additive interaction, and results less than −10 indicate an antagonistic interaction.

### 5.14 Characterization of multicellular tumor spheroids

SEM imaging: Spheroids were placed on poly‐l‐lysine‐coated plastic 12 mm round coverslips (Bar Naor, Israel) and were then fixed in 2.5% paraformaldehyde (PFA) in 0.075 M cacodylate buffer for 1 h, rinsed in cacodylate buffer, and dehydrated in a graded series of alcohols: 50%, 75%, 95% through absolute alcohol. The samples were then dried using JCP‐1 Critical Point Dryer (Denton). The coverslips were attached to SEM stubs and sputter coated with gold/palladium using a Desk IV sputter system (Denton Vacuum). Images were obtained with a Scanning Field Emission Supra 25 Scanning Electron Microscope (Zeiss).

Spheroid staining: Spheroids of SK‐136 with 3 T3 were fixed in 2% paraformaldehyde (PFA) and embedded in paraffin. Slices of 10 μm were stained with anti‐mouse Ki67 antibody (R&D), hematoxylin and eosin stain (H&E) or anti‐caveolin‐1 antibody (Cell Signaling, cat no. 3267, 1 μg/mL). H&E images were quantified in FIJI by first applying a color deconvolution to separate the images into three channels. The dominant channel was converted to grayscale, and standard thresholding analysis was used by applying the same threshold cutoff value for all the images. The amount of interstitial space per image was defined as the ratio of white space within a Region of Interest (ROI) over the total area of each ROI.

### 5.15 Statistical analysis

Experiments were conducted with a minimum of two replicates per condition, exact number of replicates is listed in relevant figure caption as n. Statistical analysis was performed using GraphPad Prism (GraphPad 10 Software). To standardize the results, a normalization procedure was performed in which each well was compared to its baseline and then adjusted relative to the control. A two‐tailed Student's unpaired *t*‐test was conducted to compare control versus treated groups. The significance level was set at *p* < 0.05, and *p* values are provided in figure captions. Error bars depict the standard deviation (SD).

## AUTHOR CONTRIBUTIONS

**Maytal Avrashami:** Conceptualization; data curation; formal analysis; investigation; methodology; validation; visualization; writing – original draft. **Danna Niezni:** Formal analysis; investigation; writing – review and editing. **Dana Meron Azagury:** Investigation; writing – review and editing. **Hagit Sason:** Writing – review and editing. **Yosi Shamay:** Conceptualization; funding acquisition; supervision; writing – original draft.

## FUNDING INFORMATION

This work was supported by the Israel Science Foundation (ISF‐901/19), and by the Foundation Deutsches Krebsforschungszentrum, Heidelberg, with the Ministry of Science, Technology & Space (DKFZ‐MOST No. 314989).

## CONFLICT OF INTEREST STATEMENT

The authors declare that they have no competing interests.

### PEER REVIEW

The peer review history for this article is available at https://www.webofscience.com/api/gateway/wos/peer-review/10.1002/btm2.10731.

## Supporting information


**Data S1.** Supplementary Materials.

**Movie S1.** Penetration profile over time imaged using LionHeart automated microscope of neratinib in Cal33 spheroids (GFP channel). Green = GFP signal.

**Movie S2.** Penetration profile over time imaged using LionHeart automated microscope of neratinib in Cal33 spheroids (GFP channel + BF channel). Green = GFP signal.

**Movie S3.** Penetration profile over time imaged using LionHeart automated microscope of afatinib in Cal33 spheroids (GFP channel). Green = GFP signal.

**Movie S4.** Penetration profile over time imaged using LionHeart automated microscope of afatinib in Cal33 spheroids (GFP channel + BF channel). Green = GFP signal.

**Movie S5.** Penetration profile over time imaged using LionHeart automated microscope of osimertinib in Cal33 spheroids (GFP channel). Green = GFP signal.

**Movie S6.** Penetration profile over time imaged using LionHeart automated microscope of osimertinib in Cal33 spheroids (GFP channel + BF channel). Green = GFP signal.

**Movie S7.** Penetration profile over time imaged using LionHeart automated microscope of neratinib in SK‐136:3 T3 co‐culture spheroids (GFP channel + BF channel). Green = GFP signal, red = RFP signal.

**Movie S8.** Penetration profile over time imaged using LionHeart automated microscope of afatinib in SK‐136:3 T3 co‐culture spheroids (GFP channel + BF channel). Green = GFP signal, red = RFP signal.

**Movie S9.** Penetration profile over time imaged using LionHeart automated microscope of osimertinib in SK‐136:3 T3 co‐culture spheroids (GFP channel + BF channel). Green = GFP signal, red = RFP signal.

## Data Availability

All data needed to evaluate the conclusions in the paper are present in the paper and/or the Supplementary Materials. Additional data related to this paper are available on request from the corresponding author.
