# Supplementary material for: Green/red fluorescent protein disrupting drugs for real‐time permeability tracking in three‐dimensional tumor spheroids
Source: Bioeng Transl Med. 2024 Dec 9;10(3):e10731. doi: 10.1002/btm2.10731 (PMC12079514; doi:10.1002/btm2.10731)
Supplement: Supplementary file 1 — Data S1. Supplementary Materials. [file BTM2-10-e10731-s005.docx]

Supplementary Materials for

**Green/red fluorescent protein disrupting drugs for real‐time permeability tracking in three‐dimensional tumor spheroids**

Maytal Avrashami *et al.*

*Corresponding author, Dr. Yosi Shamay

Email: [yshamay@technion.ac.il](mailto:yshamay@technion.ac.il)

**
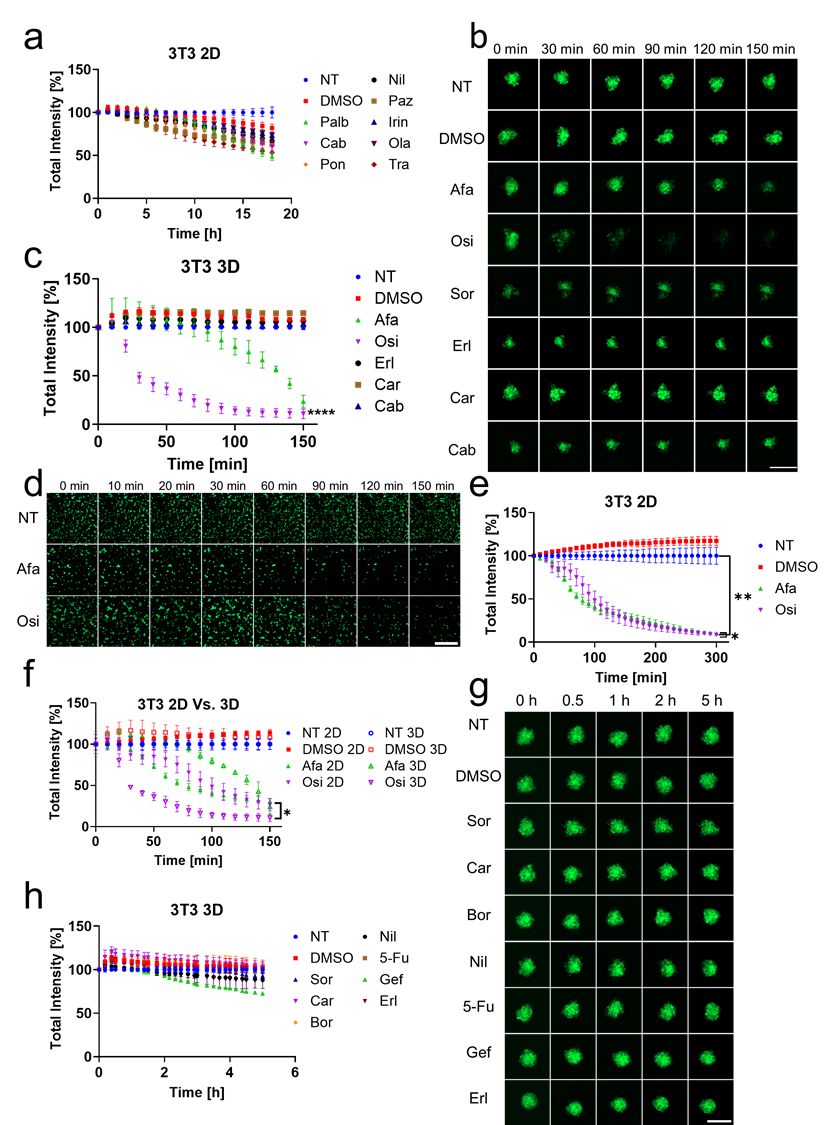
**

**Figure S1. Comparison analysis between 2D and 3D 3T3 fibroblast cells cultures.**
(a) Quantification of GFP total intensity signal of 3T3 2D cell culture in the microscope images, n = 2. (b) Representative images taken using LionHeart automated microscope of 24-h-old 3T3 3D cell culture incubated with various drugs, at a concentration of 0.03 mg/mL for 150 min. Scale bar = 250 µm, green = GFP signal. (c) Quantification of GFP total intensity signal of 3T3 3D cell culture of the microscope images. (d) Representative time lapse images taken using LionHeart of 3T3 2D cell culture incubated with 0.03 mg/mL of osimertinib or afatinib. Green = GFP signal, scale bar = 500 µm. (e) Quantification of GFP total intensity signal of 3T3 2D cell culture of the microscope images, n = 4.
(f) Comparison between the GFP total intensity signal of 3T3 cells incubated with afatinib or osimertinib in 2D (full symbols, n = 4) or 3D cultures (hollow symbols, n = 3). (g) Representative time lapse images taken using LionHeart of 24-h-old 3T3 3D cell culture incubated with 0.03 mg/mL of various drugs for 5 h. Green = GFP signal, scale bar = 250 µm. (h) Quantification of GFP total intensity signal of 3T3 2D cell culture of the microscope images, n = 4. 5-Fluorouracil (5-Fu). Error bars indicate mean ± SD, *p < 0.05, **p < 0.01, ****p < 0.0001 by unpaired t-test.


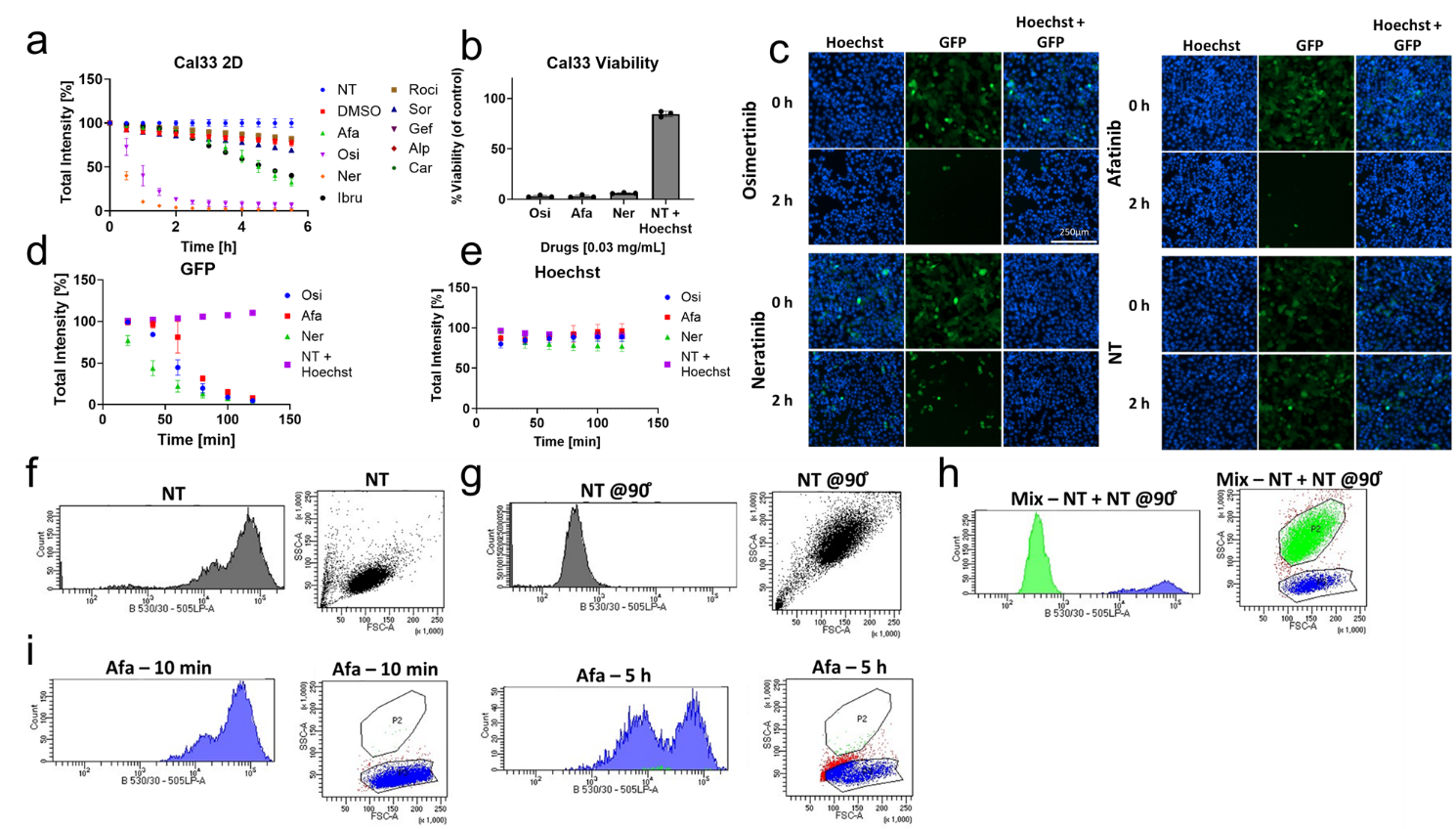


**Figure S2. Evaluating the effect of GFP disrupting drugs on Cal33 2D cell cultures through various assays.** (a) Quantification of GFP total intensity signal of Cal33 2D cell culture from the microscope images, n = 3. (b) CTG 2D results of Hoechst stained Cal33 2D cell culture after 5 h of incubation with the drugs at a concentration of 0.03 mg/mL, n = 3. (c) Representative image taken using LionHeart automated microscope of Hoechst staining of Cal33 cells incubated with 0.03 mg/mL osimertinib, neratinib or afatinib for 2 h. Green = GFP signal, Blue = Hoechst 33342.
(d, e) Quantification of GFP (d) and Hoechst (e) total intensity signal of Cal33 2D cell culture in the microscope images, n = 3. (f-i) FACS results of non-treated live (f), non-treated dead (g), a mixture of both (h) and afatinib treated (i) Cal33 cells. Results present count vs. Intensity histograms with 530/30 filter and Forward Scatter-A/Side Scatter-A dot plots, gates are set as P1 for live cells (blue), P2 for dead cells (green).


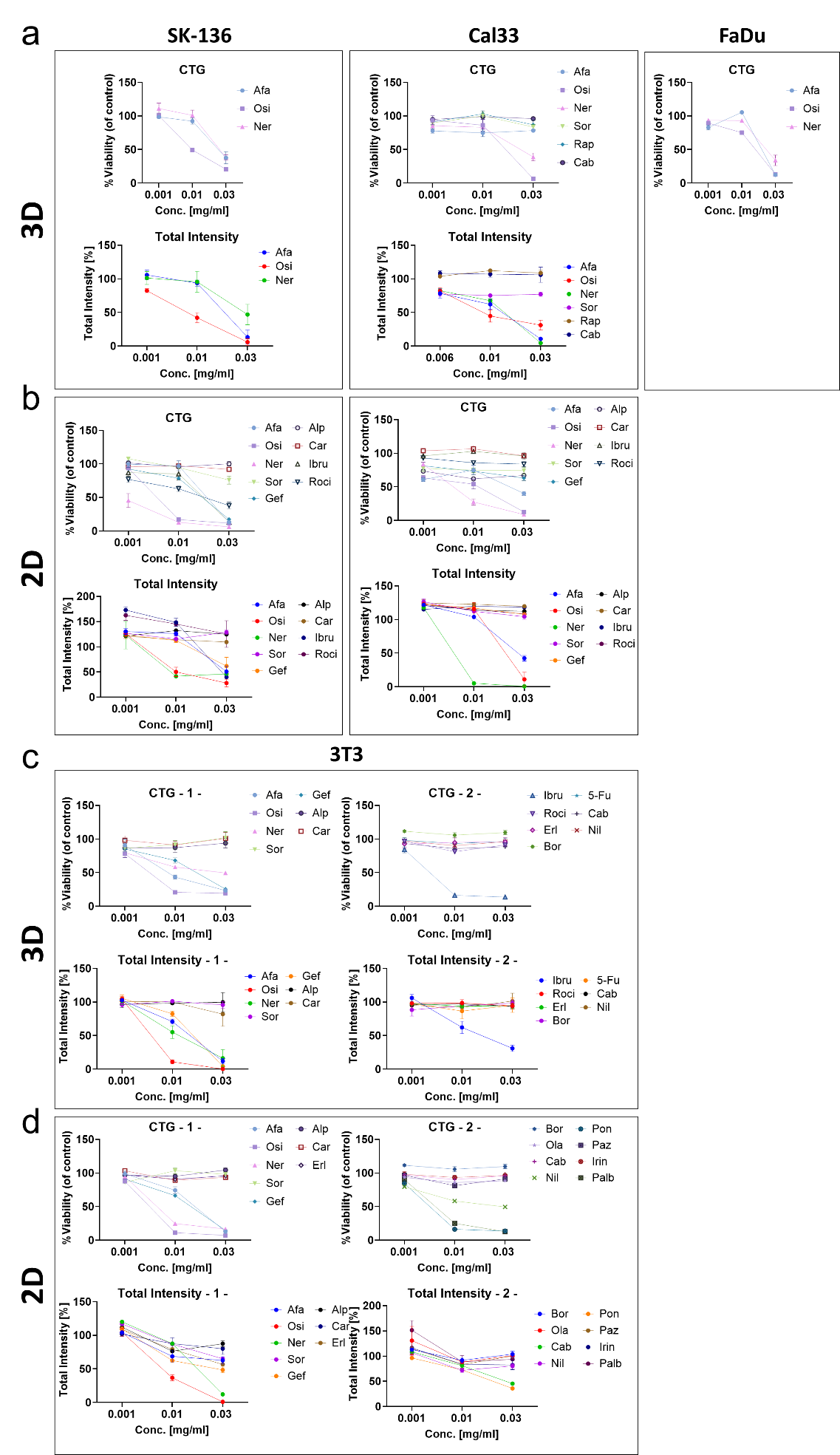


**Figure S3. Cell viability assay in 2D and 3D cell cultures across various cell lines used in the study compared with total intensity values at different concentrations.** All cell lines were incubated with 3 concentrations of various drugs 0.001, 0.01, 0.03 mg/mL for 5 h. CTG 2D (a) and 3D (b) results for SK-136 (left), Cal33 (middle) and FaDu (right) are presented on the top of each box and on the bottom are the relevant quantification of GFP and RFP total intensity signals. CTG 2D (c) and 3D (d) results for 3T3 are presented on the top of each box and on the bottom are the relevant quantification of GFP total intensity signals.

**
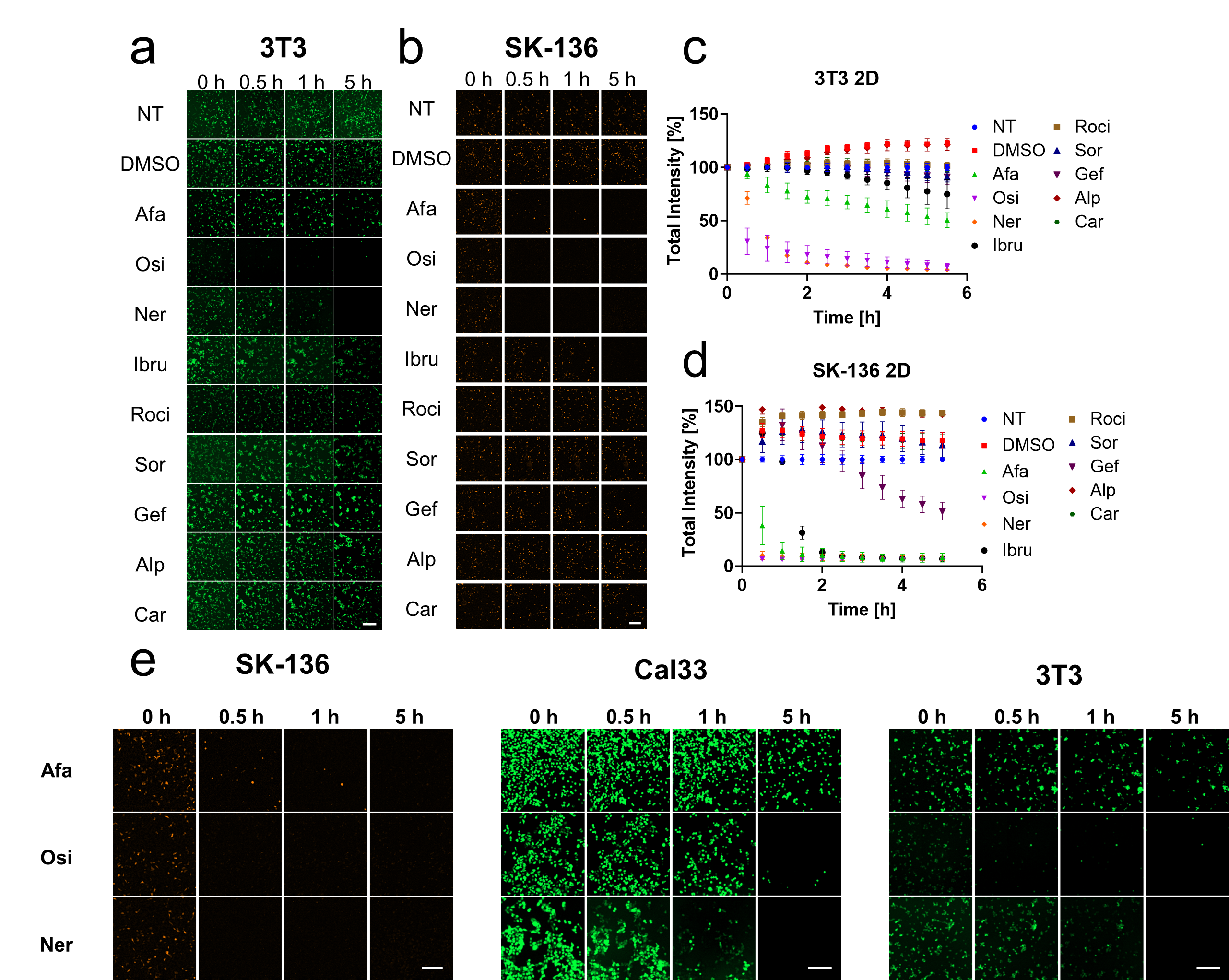
**

**Figure S4. Additional screening with drugs containing acrylamide moieties and covalent warheads in various cell lines.** (a, b) Representative time lapse images taken using LionHeart automated microscope of 3T3 (a) and SK-136 (b) 2D cell cultures, incubated with various drugs at a concentration of 0.03 mg/mL for 5 h. Scale bar = 250 µm, green = GFP signal, red = RFP signal. (c, d) Quantification of GFP/RFP total intensity signal of 3T3 (c) and SK-136 (d) 2D cell cultures from the microscope images, n = 3. Error bars indicate mean ± SD. (e) Representative time lapse images taken using LionHeart of SK-136 (left), Cal33 (middle) and 3T3 (right) 2D cell culture, incubated with afatinib, osimertinib and neratinib for 5 h. Scale bar = 250 µm, green = GFP signal, red = RFP signal.

**
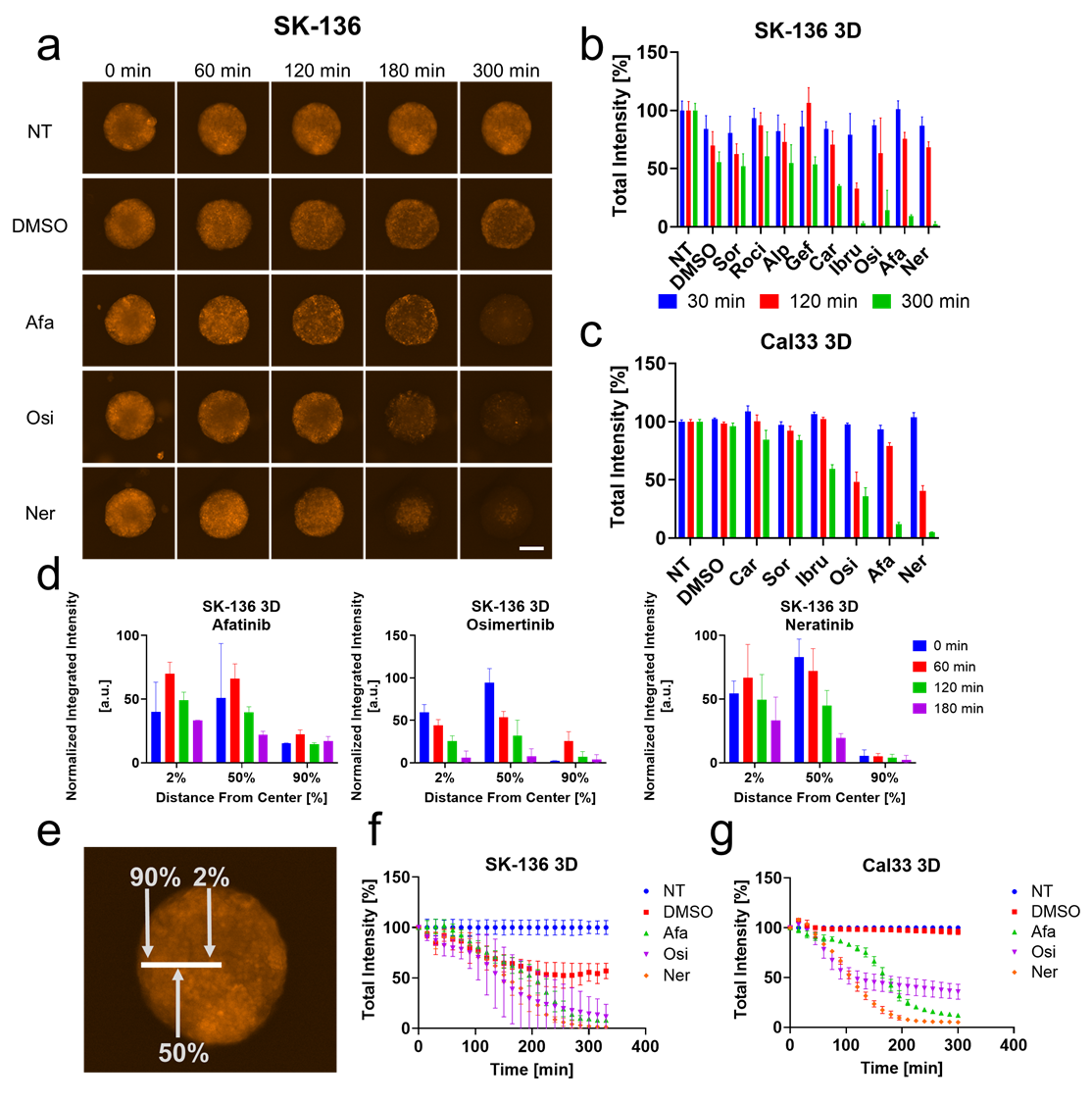
**

**Figure S5. Drug screening on Cal33/SK-136 spheroids and penetration profile of different drugs on SK-136 spheroid model.** (a) Representative images taken using LionHeart automated microscope of SK-136 3D spheroids incubated with 0.03 mg/mL of afatinib, osimertinib and neratinib for 5 h.  Red = RFP signal, scale bar = 200 µm. (b, c) Quantification of RFP/GFP total intensity signal of SK-136 (b, n = 3) and Cal33 (c, n = 4) spheroids incubated with different drugs for 5 h, at 3 time points. (d, e) Radial intensity values at different time points for SK-136 spheroids treated with afatinib (left), osimertinib (middle) and neratinib (right), as a function of the distance from the center, as depicted schematically in (e), n = 2. (f, g) Quantification of RFP/GFP total intensity signal of SK-136 (f, n = 3) and Cal33 (g, n = 4) spheroids incubated with afatinib, osimertinib and neratinib for 5 h. Error bars indicate mean ± SD.


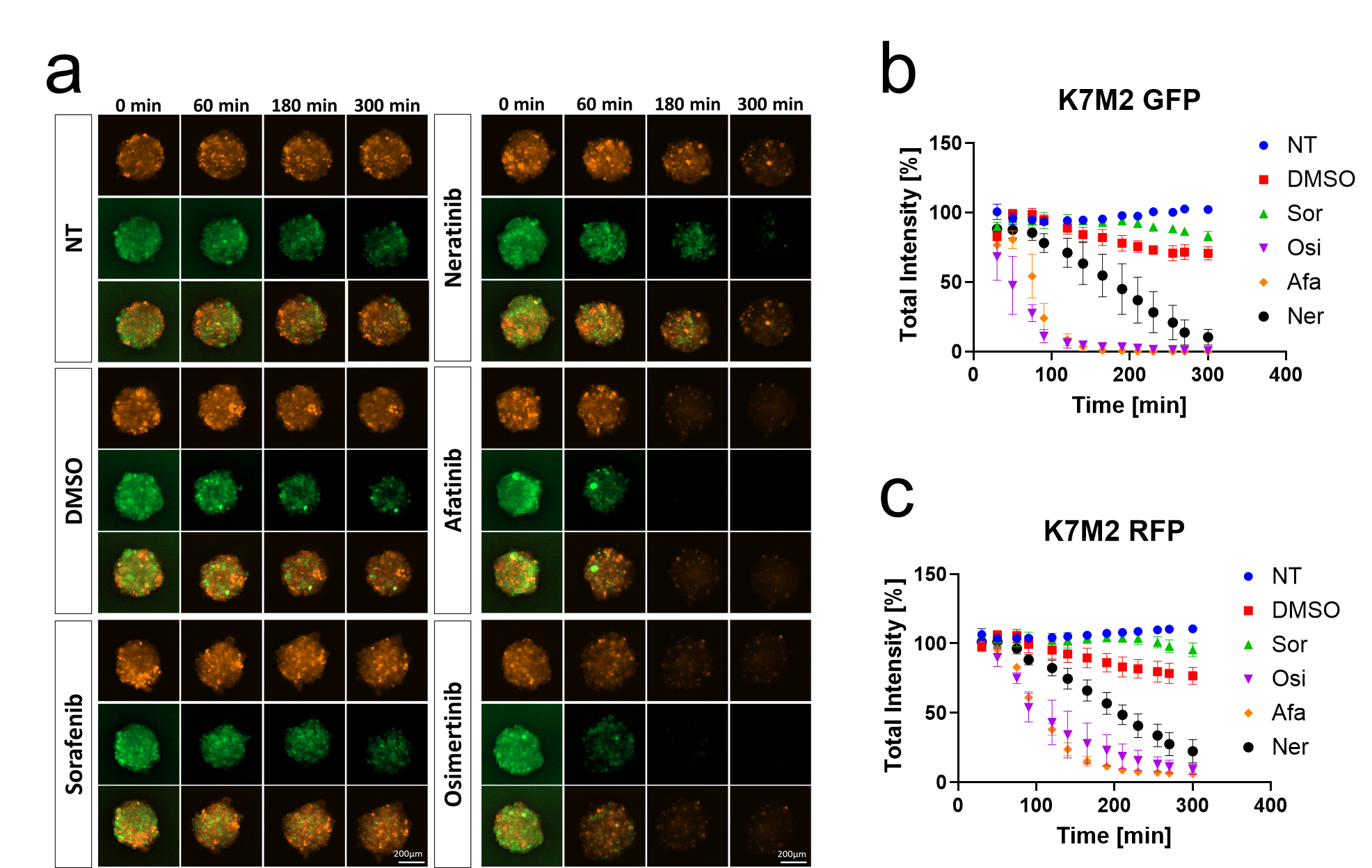


**Figure S6. Effect of GFP disrupting drugs on K7M2 spheroids expressing both GFP and RFP.** (a) Representative images taken using LionHeart automated microscope of K7M2 spheroids incubated with DMSO, sorafenib, neratinib, afatinib and osimertinib at a concentration of 0.03 mg/mL for 300 min. Green = GFP signal, red = RFP signal, scale bar = 250 µm. (b, c) Quantification of GFP (b) and RFP (c) total intensity signal of K7M2 spheroid incubated with various drugs for 300 min, compared to non-treated spheroids (NT). Error bars indicate mean ± SD, n = 3.

**Figure S7. Stability of GFP fluorescence in different experiments.** Quantification of GFP total intensity signal of 96-h-old Cal33 spheroids from different experiments, n > 72.


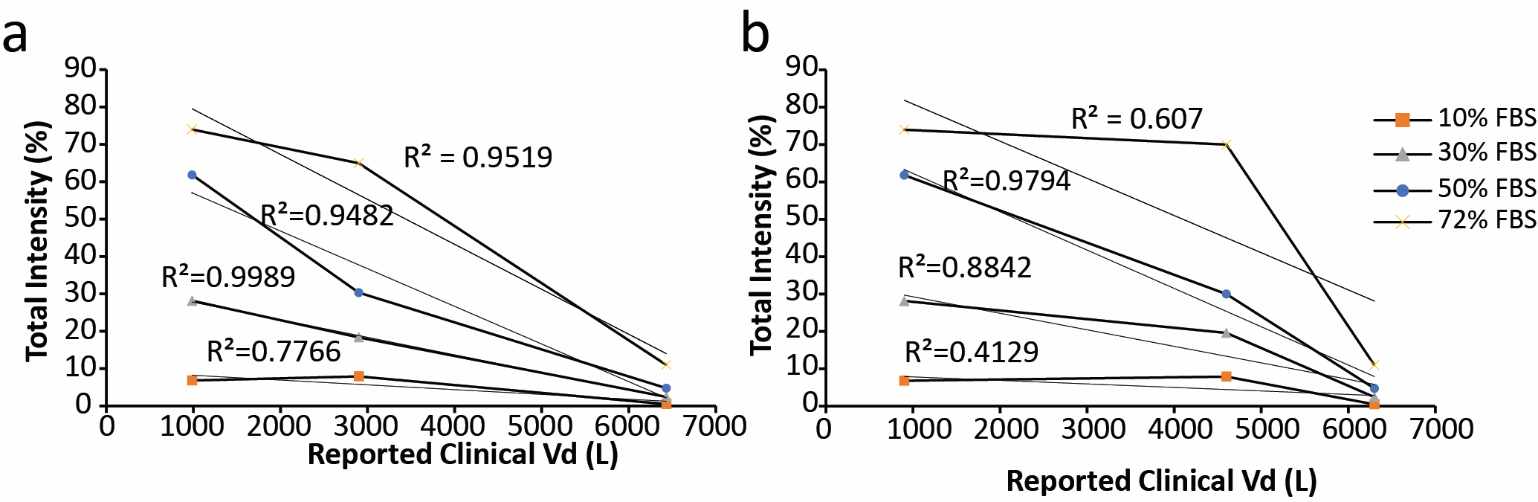


**Figure S8. Correlation plot to reported clinical volume of distribution values.** (a) Vd values derived from 3 recent papers (> 2017). (b) Vd values of afatinib for the first phase 1 clinical trial in 2011 which values were not reproduced.


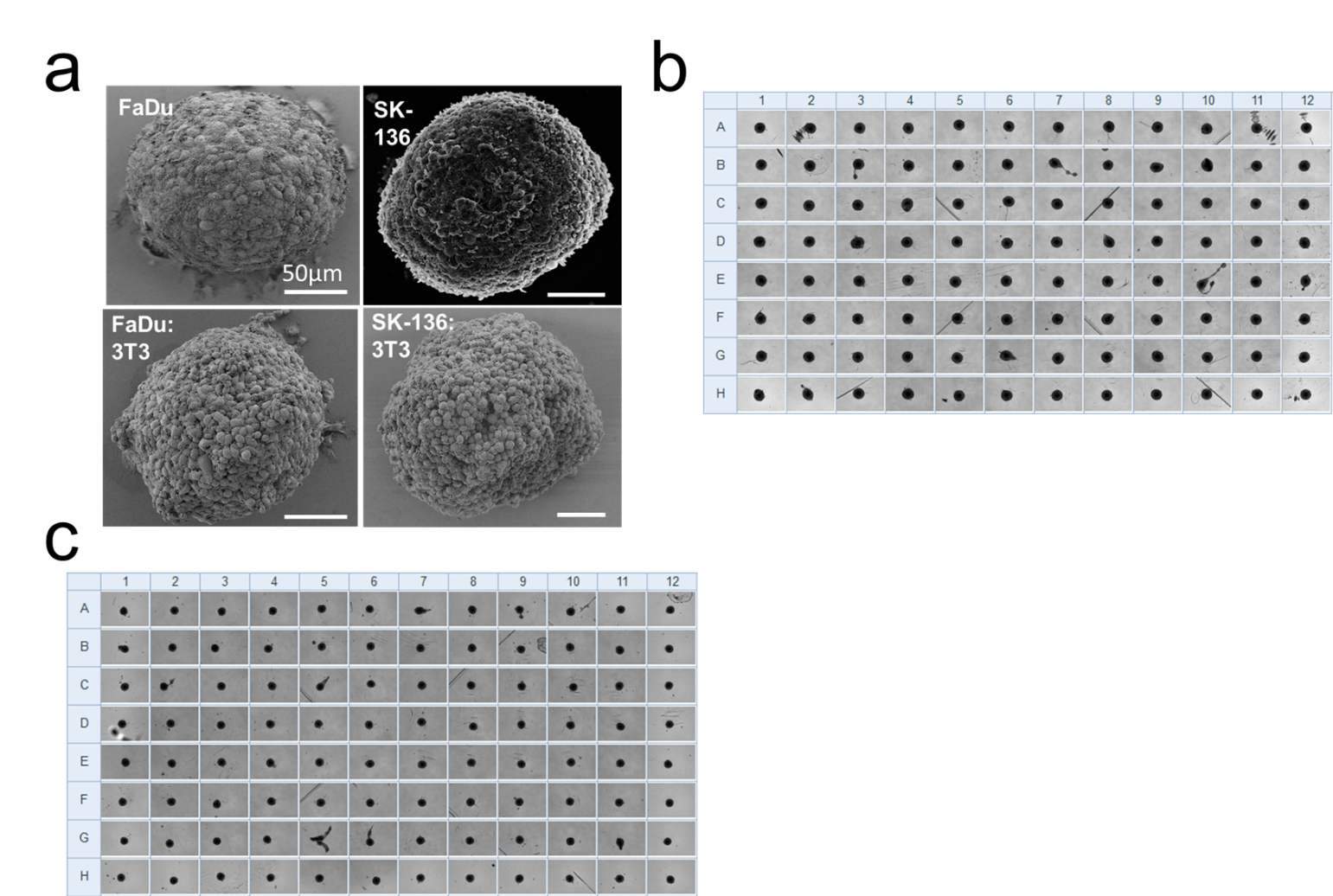


**Figure S9. Additional characterization of co-culture spheroid model.** (a) HR-SEM images of FaDu spheroids (left upper panel), SK-136 spheroids (right upper panel), FaDu:3T3 co-culture spheroids (left bottom panel) and SK-136:3T3 co-culture spheroids (right bottom panel). Scale bars = 50µm.
(b, c) Representative images of 96-ULA well plates with co-culture of 1000:500 cells per well of FaDu:3T3 (b) or 500:250 cells per well of SK-136:3T3 (c) 96 h after seeding, showing homogenous spheroids size.


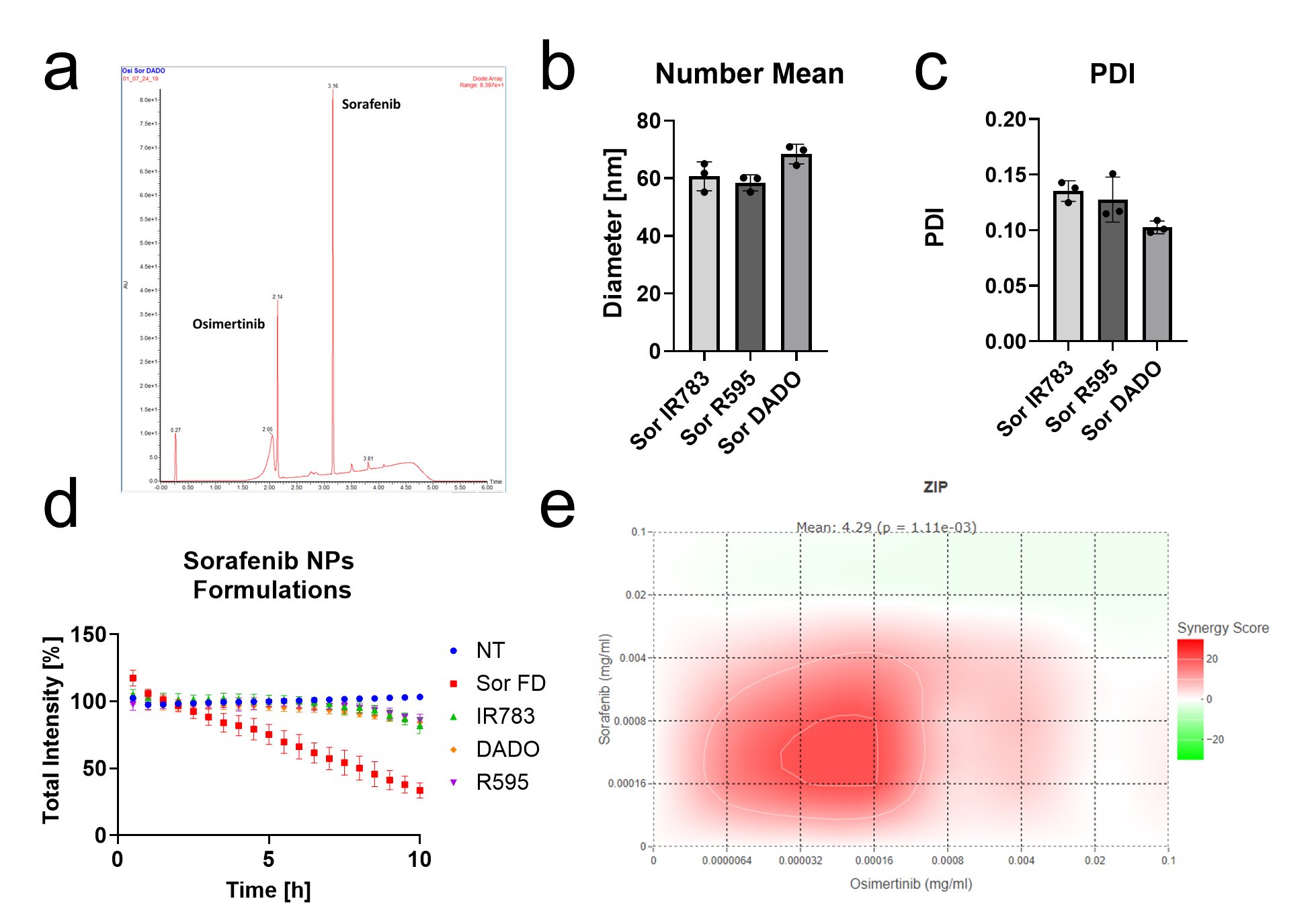


**Figure S10. Nanoparticles characterization and the effect of sorafenib nanoparticles on the GFP fluorescence of Cal33 spheroids.** (a) Representative HPLC result of sorafenib-osimertinib dual drug nanoparticles with PDA-PDO-In820 (DADO) as stabilizer. (b, c) DLS measurements of the different sorafenib nanoparticles diameter (b) and poly dispersity index (PDI) (c), n = 3. (d) Quantification of GFP total intensity signal of Cal33 spheroids incubated with sorafenib nanoparticles for 10 h, compared with sorafenib as free drug, n = 4. Error bars indicate mean ± SD. (e) Synergy calculation of osimertinib and sorafenib in Cal33 cells, presented as a heat map.

**Movie S1.**

Penetration profile over time imaged using LionHeart automated microscope of neratinib in Cal33 spheroids (GFP channel). Green = GFP signal.

**Movie S2.**

Penetration profile over time imaged using LionHeart automated microscope of neratinib in Cal33 spheroids (GFP channel + BF channel). Green = GFP signal.

**Movie S3.**

Penetration profile over time imaged using LionHeart automated microscope of afatinib in Cal33 spheroids (GFP channel). Green = GFP signal.

**Movie S4.**

Penetration profile over time imaged using LionHeart automated microscope of afatinib in Cal33 spheroids (GFP channel + BF channel). Green = GFP signal.

**Movie S5.**

Penetration profile over time imaged using LionHeart automated microscope of osimertinib in Cal33 spheroids (GFP channel). Green = GFP signal.

**Movie S6.**

Penetration profile over time imaged using LionHeart automated microscope of osimertinib in Cal33 spheroids (GFP channel + BF channel). Green = GFP signal.

**Movie S7.**

Penetration profile over time imaged using LionHeart automated microscope of neratinib in SK-136:3T3 co-culture spheroids (GFP channel + BF channel). Green = GFP signal, red = RFP signal.

**Movie S8.**

Penetration profile over time imaged using LionHeart automated microscope of afatinib in SK-136:3T3 co-culture spheroids (GFP channel + BF channel). Green = GFP signal, red = RFP signal.

**Movie S9.**

Penetration profile over time imaged using LionHeart automated microscope of osimertinib in SK-136:3T3 co-culture spheroids (GFP channel + BF channel). Green = GFP signal, red = RFP signal.
